# On the occasion of the centennial of the Nobel Prize in Physiology or Medicine, 1923: Nicolae C. Paulescu—between scientific creativity and political fanatism

**DOI:** 10.1007/s00592-023-02136-6

**Published:** 2023-07-05

**Authors:** Alberto de Leiva-Hidalgo, Alejandra de Leiva-Pérez

**Affiliations:** 1 Universitat Autònoma de Barcelona, Bellaterra, Spain; 2 Fundació DIABEM, Barcelona, Spain

**Keywords:** Nobel Prize, Controversy, Antidiabetic hormone, antisemitism, Pancreina, Insulin

## Abstract

**Aims:**

Since the Nobel Prize in Physiology or Medicine was awarded in 1923 to FG Banting and JJR Macleod, many voices have been raised against this decision. The bitterest protest was that of the Romanian scientist Nicolae C. Paulescu. In 2002, The Romanian Academy of Sciences, the European Association for the Study of Diabetes (EASD) and the International Diabetes Federation (IDF) planned to hold a series of academic events the following year in Paris to acknowledge Paulescu's scientific merits in the discovery of the antidiabetic hormone. However, the initiative was cancelled in August 2003, when the European Center of the Simon Wiesenthal Foundation (SWC) accused Paulescu of being antisemitic. The authors of this manuscript have decided to approach "the Paulescu case" from its double aspect, scientific and sociopolitical, to analyze the circumstances surrounding the discovery of the antidiabetic hormone, and Paulescu's alleged antisemitic past in the historical context of the Romanian nation in the interwar period.

**Methods:**

We contacted the SWC and people related to the 2003 events in Paris. We performed a comparative review of the documents published by the Toronto group and by Paulescu and analyzed the correspondence and articles generated by international experts from the scientific community interested in the controversy. We carried out an exhaustive bibliographic search through several online catalogs (INDEXCAT, NLM Gateway, EUREKA, MEDHIST). We travelled to Bucharest, where we visited Paulescu's house-museum, interviewed a former student of the Romanian professor, and a prominent medical historian who was knowledgeable about Paulescu's scientific and political biography. Dan Angelescu†, son of Dr. Constantin Angelescu (1904–1990), Paulescu's nephew and collaborator, provided us with a copy of all the available documentation from Paulescu's personal archive. It constitutes an essential source for understanding Paulescu's personal, political and academic biography. *Archives consulted:* Românǎ Academy (Bucharest). Personal Archive of Paulescu, House -Museum (Bucharest)*. Romanian Jewish Heritage (Bucharest). http://romanianjewish.org/ **. Simon Wiesenthal Center (Los Angeles, CA) http://www.wiesenthal.com **. Romanian Patent Office. Oficiul de Stat pentru Invenții şi Mǎrci (OSIM) (Bucharest)***. Nobel Archives (Stockholm) https://www.nobelprize.org. Internet Archive (San Francisco, CA) https://archive.org **. Wellcome Library (London) https://wellcomelibrary.org **. The European Library https://www.theeuropeanlibrary.org/ **. US National Library of Medicine, NLM historical collections http://www.nlm.nih.gov/hmd/index.html **. US. Holocaust Memorial Museum http://www.ushmm.org/ (*: archive consulted on site; **: material found in the online catalog of the archive; ***: archivists sent us digitized copies of archival material). *Books consulted for information on the history of Romania and antisemitism:* “Nationalist ideology and antisemitism. The case of Romanian intellectuals in the 1930s”, by Leon Volovici; “The mystique of ultranationalism: History of the Iron Guard, Romania, 1919–1941” by Francisco Vega; “Romania 1866–1947”, by Keith Hitchins; “History of Romania. Compendium”, by Ioan-Aurel Pop and Joan Bolovan; “The Holocaust in Romania. The destruction of Jews and Gypsies under the Antonescu regime, 1940–1944”, by Radu Ioanid; “The Jews of East Central Europe between the World Wars”, by Ezra Mendelson; “Cultural Politics in Greater Romania. Regionalism, Nation Building and Ethnic Struggle, 1918–1930”, by Irina Livezeanu, and “Judeophobia. How and when it is born, where and why it survives”, by Gustavo Daniel Perednik. Articles are referenced in the bibliography section at the end of the manuscript.

**Results:**

A-Nicolae Paulescu developed an intense long-term research activity, which included complete pancreatectomy and preparation of a pancreatic extract (PE) containing the antidiabetic hormone he called pancreina. Parenteral administration of the PE achieved excellent results in the treatment of experimental diabetes in dogs and induction of hypoglycemia in the healthy animal. This work was initiated before 1916 and published at least eight months antedating the publication of the first article by Banting and Best (February 1922), who were acquainted with Paulescu's results, but misinterpreted them. The pancreatic extract of the two Canadian researchers, -iletin/insulin-, only achieved similar results to that of the Romanian scientist once they abandoned the use of the "degenerated pancreas" extract (ligation of the ductal system), replacing it with the pancreas of adult or fetal bovine. Pancreina and insulin were very similar. The award of the Nobel Prize in Physiology or Medicine to FG Banting and JJR Macleod in October 1923 honored the successful clinical use of insulin in patients with diabetes mellitus. Paulescu's achievements were ignored. B-Nicolae Paulescu publicly manifested his Judeophobic ideology on multiple occasions in academic and political interventions and in publications and participated with other figures from the Romanian intellectual sphere in the founding of the Uniunea Național Crestinǎ (UNC, National Christian Union) in 1922 and of the Liga Apǎrǎrii Național Cresține (LANC, League for Christian National Defense) in 1923, antisemitic far-right political parties, associated with an irrational Christian orthodoxy and hatred of Jews. Paulescu played a pivotal role in the spread of antisemitism.

**Conclusions:**

A-The Romanian scientist NC Paulescu started an intense research program aimed at the isolation of the antidiabetic hormone before 1916, including an original procedure of pancreatectomy in the dog and the elaboration of a pancreatic extract that achieved excellent results in the treatment of experimental diabetes, demonstrating its beneficial effects on the metabolism of carbohydrates, proteins and fats and reducing both glycosuria and glycemia and the urinary excretion of ketone bodies of depancreatized dogs toward normality. The results of these investigations were published in 1920 and 1921, predating the first report published by FG ​​Banting and CH Best in February 1922. It has been sufficiently demonstrated that Canadian researchers were aware of Paulescu's excellent results, mentioning them only in passing, albeit erroneously misrepresenting key results of the Romanian scientist's publication in the aforementioned seminal Canadian article. Expert historians and international scientists have recognized that the pancreatic extract that Paulescu called pancreina and that obtained by Banting and Best, insulin, were very similar. The October 1923 award of the Nobel Prize in Physiology or Medicine to FG Banting and JJR Macleod ignored Paulescu's scientific achievements in the treatment of experimental diabetes and rewarded the extraordinary advance of insulin treatment in human diabetes. B-At the end of August 2003, a few days before the date of the celebration at the Hôtel Dieu in Paris of the scheduled program of tribute to the scientific merits of NC Paulescu and his important contribution to the discovery of the antidiabetic hormone, convened by the Romanian Academy and the International Diabetes Federation, the Wiesenthal Foundation publicly accused the Romanian scientist of being an antisemite, an act that determined the cancellation of the announced events. The exhaustive investigation of the personal convictions and antisemitic behavior of Nicolae C. Paulescu has undoubtedly documented the Judeophobic ideology of the Romanian scientist, linked to his orthodox religious radicalism, manifested in multiple documents (mostly pamphlets) and interventions in collaboration with other relevant personalities of the Romanian intelligentsia of his time. Furthermore, Paulescu participated in the creation of political organizations of the most radical extreme right that played a fundamental role in the spread of antisemitism amongst the Romanian population and the university community.

**Supplementary Information:**

The online version contains supplementary material available at 10.1007/s00592-023-02136-6.

## Resurgence of interest in Paulescu's contribution to the discovery of the antidiabetic hormone (1968–2003)

**Fig. 1:**
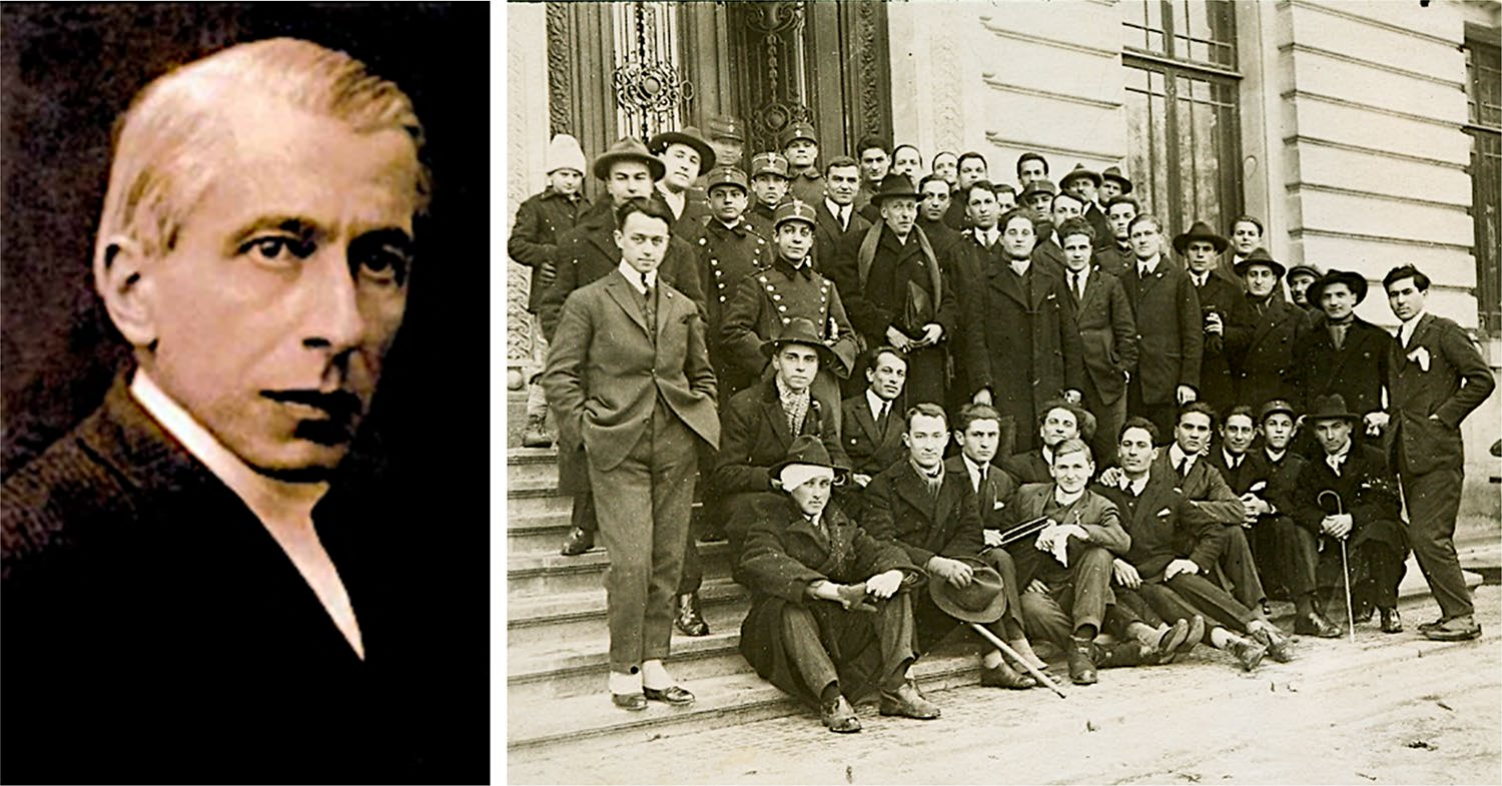
Left: Portrait of Nicolae Constantin Paulescu. Author and date unknown. https://commons.wikimedia.org/w/index.php?title=File:Nicolae_Paulescu.Foto02.jpg&oldid=651946579. Right: N.C. Paulescu and his students on the access stairs to the Faculty of Medicine, University of Bucharest. Year 1926. Paulescu's personal archive. Courtesy of Dan Angelescu†

Nicolae C. Paulescu died on July 7, 1931, from kidney complications related to a bladder carcinoma. With the tragic vicissitudes of the Second World War and the rise to power in Romania of the Communist Party, Paulescu's traces were erased from the history of Romanian science (Fig. 1) [1].

The renaissance of Nicolae Constantin Paulescu in the scientific community and his contribution to the discovery of the antidiabetic hormone have been mainly the result of the intense and continuous work of Ion Pavel, Ian Murray, Constantin Ionescu-Tirgoviste and members of the Romanian Academy.

**Fig. 2:**
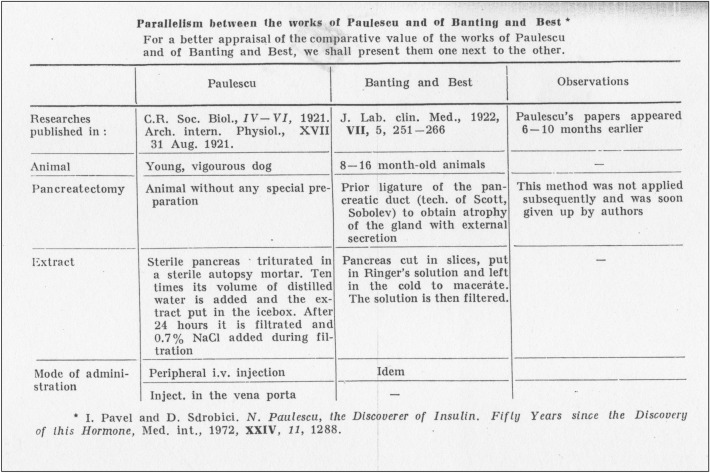
Comparison between Paulescu's pancreatic extract (1921) and Banting and Best'(1922) [2]

*Ion Pavel* (1897–1992) was a student of Paulescu in the academic year 1916–1917. Pavel, for many years professor at the Clinic for Nutritional Diseases and Diabetes at Cantacuzino Hospital, is considered the founder of Romanian diabetology. In 1939, he started the Romanian Diabetes Registry and in 1944 he published the monograph “Le Diabète”, which earned him the prize of the Paris Academy of Sciences. A member of the Romanian Academy and the French Academy of Medicine, for many years he vindicated Paulescu's priority in the discovery of the antidiabetic hormone, publishing two monographs and a series of articles on the subject (Fig. 2) [2–4].

*Ian Murray* (1899–1974), Professor of Physiology at Anderson College of Medicine in Glasgow, Vice-President of the British Diabetic Association and founding member of the International Diabetes Federation (IDF) found convincing evidence that several researchers had obtained pancreatic extracts with greater or lesser biological activity, capable of generating symptomatic relief of diabetes in pancreatectomy dogs, prior to the first experiences in Toronto by Frederick G. Banting and Charles H. Best. Of all of these pioneering contributions, the most significant, according to Murray, was that of Nicolae C. Paulescu, whom he considered the discoverer of the pancreatic hormone, now known as insulin.

The Scottish professor wrote in October 1968 to the Professor of Physiology (then Grigore Benetato) at the Faculty of Medicine, University of Bucharest. In the absence of a response, Murray contacted Professor Ion Pavel, a member of the Romanian scientific community with recognized prestige among IDF members. In this way, a cordial correspondence between Murray and Pavel was established on November 17, 1968, which lasted until 1974. The documents sent by Murray to Pavel included three publications that confirmed the international growing interest in Paulescu's work, and the suggestion to generate a Romanian delegation to participate in the IDF general council meeting to be held at the VII Congress in Buenos Aires of the IDF (1970) to debate research achievements related to the discovery of insulin [5–7].

**Table:** 

*“*The fascinating story of the discovery of insulin still provokes controversy (…). Insufficient recognition has been given to Paulescu, the distinguished Romanian scientist, who at the time when the Toronto team were commencing their research, had already succeeded in extracting the antidiabetic hormone of the pancreas and proving its efficacy in reducing the hyperglycaemia in diabetic dogs (…). His results, published in August 1921, proved convincingly that he had succeeded in isolating the antidiabetic hormone of the pancreas and demonstrating its action in lowering the blood sugar in both diabetic and normal dogs (…). Banting and Best are commonly believed to have been the first to have succeeded in isolating insulin. They have been hailed as their ‘discoverers’. Their work, however, may more accurately be construed as confirmations of Paulescu’s findings (…). There can be no doubt that pancreina and iletin were identical. Unfortunately, both these extracts caused such local irritation when injected subcutaneously that administration by this route was impossible.”
Ian Murray, 1971 [7]

On February 29, 1972, Murray wrote to Pavel in these terms: “My suggestion is that the IDF should institute a Paulescu Memorial Lectureship, for which relevant scientists would be selected at future congresses of the federation [1; p. 593].

**Fig. 3:**
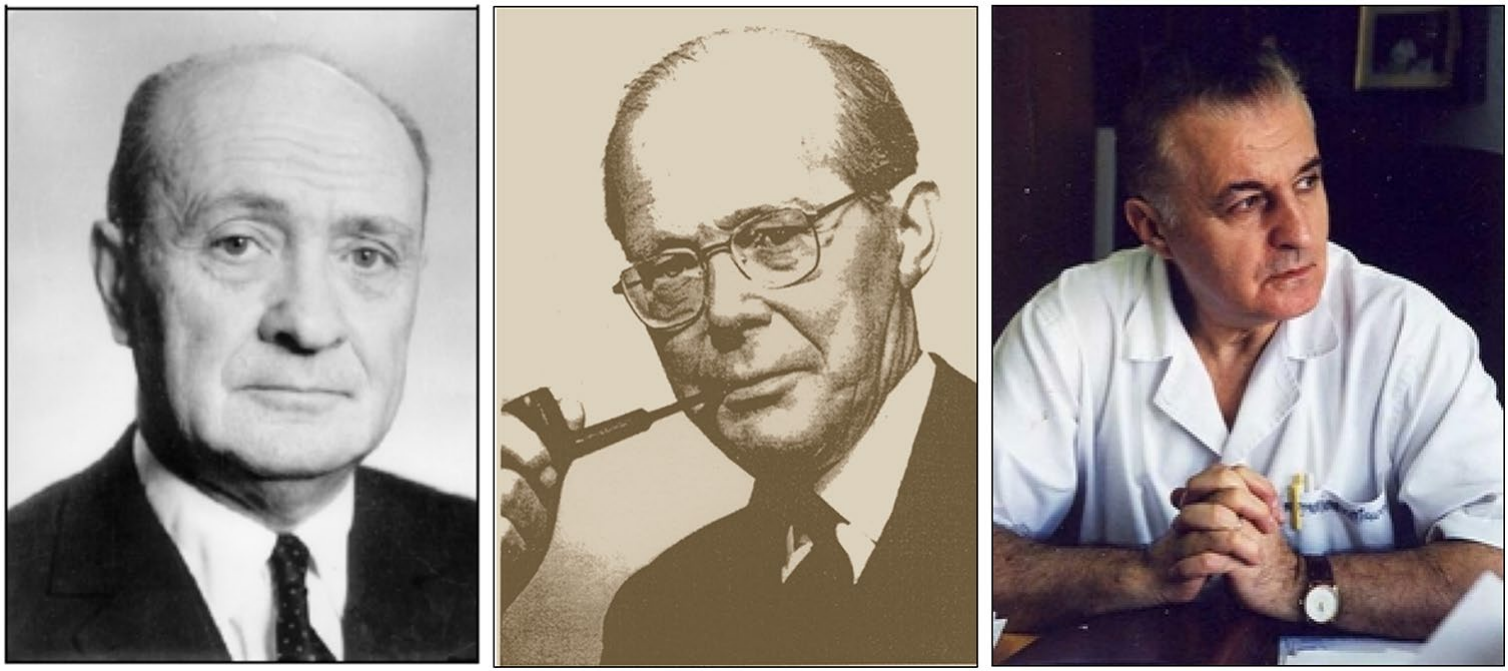
From left**:** Portraits of Ion Pavel, Ian Murray and Constantin Ionescu-Tirgoviste, who played a key role on the renaissance of Nicolae Constantin Paulescu in the scientific community and his contribution to the discovery of the antidiabetic hormone

*Constantin Ionescu-Tirgovişte* was born in Târgovişte in 1937. University professor since 1999 at the Faculty of Medicine, University of Bucharest, he also directed between 1997 and 2007 the University Clinic for Diabetes, Nutrition and Metabolic Diseases and the National Institute of Diabetes, Nutrition and Metabolic Diseases "Nicolae C. Paulescu" in Bucharest. A full member of the Romanian Academy, Ionescu-Tirgoviste has continued with extraordinary interest the work initiated by I. Murray and Ion Pavel to claim Paulescu's priority in the discovery of the antidiabetic hormone (Fig. 3) [8–11].

## IDF Report of the Special Committee set up to present a written summary of work leading up to the discovery of insulin, 1971

In the VII Congress of the International Diabetes Federation celebrated in Buenos Aires (August 1970), a Special Committee was created to devise a summary on the research developments related to the discovery of insulin. The Committee was formed by: *F. G. Young* (UK), President of the Committee; he was elected President of the IDF at the end of the Buenos Aires Congress and was Best’s personal friend. The other members of the Committee were: R. Haist (Canada), who worked with Best, and succeeded him to the Chair of Physiology at the University of Toronto; W. J. H. Butterfield (UK); Rolf Luft (Sweden) and P. Rambert (France). More information on the scientific biography of FG Young is available in Supplementary Material (1).

The report was published in 1971 (forty-nine years after the discovery of insulin). It started with a foreword from Young, and an introduction that said:

…There was no intention to detract in any way from the contributions of Banting, Best and Macleod in Toronto in 1921-1922 but rather to pay tribute to others whose published observations formed part of the background in which the investigations of the group in Toronto began fifty years ago.

The report stressed above all the importance of the clinical application of insulin carried out by the Toronto team, without paying too much attention to the importance of the physiological step. Therefore, the work of Paulescu and the other pioneers was diminished and the chronology of events was ignored. Some extracts of the report are reproduced below [12] (Supplementary Figures S1, S2).

**Table:** 

"If the isolation of a substance involves the preparation of it in pure form, as indeed the word isolation does imply, Banting and Best did not isolate insulin. What they did was to produce for the first-time pancreatic extracts containing that substance which were suitable for subcutaneous injection into animals and man, such treatment being highly effective in controlling the symptoms of diabetes mellitus in diabetic dogs and human patients."
"There can be little doubt that Paulesco, as well as Banting and Best, obtained a pancreatic extract which contained insulin, and that the pancreina and the insulin present in the crude extracts in which the hormone was first obtained, are the same substance."
"Undoubtedly, Professor N.C. Paulesco should be given special credit for the successes with which his experimental observations were crowned. But more than experimental physiology was needed if insulin was to become available in the form, and on the scale, in which it was quickly needed for the therapeutic use. The resources required involved not only the large-scale production of material of a refinement that ensured no irritant reaction on subcutaneous injection into a human being, but also the biological standardization of the hormone and the extensive clinical testing of the standardized product."
"The Nobel Prize that was given to Banting and Macleod was awarded for work which the Professorial Staff of the Caroline Institute considered to be of great importance, theoretically and practically. The practical importance of the investigations initiated by Banting and Best is witnessed by the fact that since 1922 countless numbers of diabetic people have been able to live normal lives."

Pavel corresponded with FG Young to defend Paulescu's priority. In one of these letters, Young openly explained to Pavel his personal relationship with the Toronto researchers, as well as his attempts to be as objective as possible in the contents of the IDF official report [13], [Supplementary Material (2)].

## Michael Bliss, Jasbir Singh Bajaj and John Waller recognized the priority of Paulescu in the discovery of the antidiabetic hormone antedating insulin

### Michael Bliss (1941–2017)

*Bliss*, Professor of History at the University of Toronto, wrote in 1993: “The Paulescu case was based on the realization that, in fact, Banting and Best had not produced results more impressive than Paulescu’s.” [14].

### Jasbir Singh Bajaj (1936–2019)

*J. S. Bajaj*, Professor and Director of the Department of Medicine at All India Institute of Medical Science, Health Minister (1991–98) [15], Doctor Honoris Causa from the Karolinska Institute and IDF President (1985–1988), vindicated Paulescu in his monograph *The Discovery of Insulin. A critique* [16].

### John Waller (1972-)

**Fig. 4:**
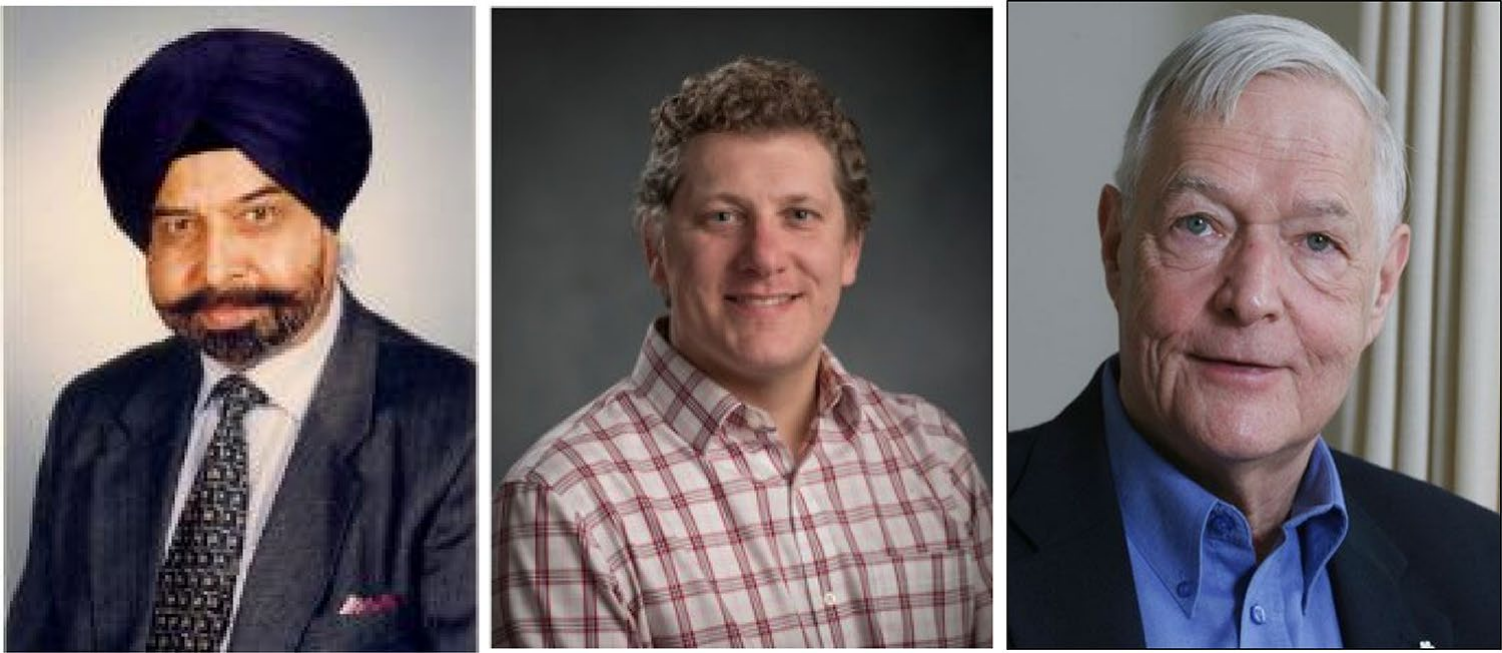
From left**:** Portraits of Jasbir Singh Bajaj (National Academy of Medical Sciences, India), John Waller and Michael Bliss (Public domain)

*John Waller*, Lecturer in the History of Medicine, University of Melbourne, wrote in his book *Fabulous Science* (Fig. 4) [17] that: “NC Paulescu published the results of his experiments involving depancreatized dogs before Banting and Best had submitted their first paper in 1921 (…). The implications were clear, if controlling the blood sugar levels of dogs was adjudged the basis of the discovery, Paulescu’s priority became extremely hard to deny”.

## Institutional acts of recognition and posthumous tribute to Paulescu (1990–2002)

In the last decade of the twentieth century, multiple acts of recognition and homage to Paulescu were held in Romania. In 1990 Paulescu was posthumously appointed a member of the Romanian Academy. On March 3, 1993, the Bucharest Institute of Diabetes, Nutrition and Metabolic Diseases adopted the name “Institulul de Diabet, Nutritie si Boli Metabolice N,C. Paulescu”. On June 27 of the same year, in Cluj-Napoca, on the occasion of the commemoration of World Diabetes Day, a postmark was issued in his honor and, in 1994, he was portrayed in one of seven stamps in memory of Romanian celebrities [18].

**Fig. 5:**
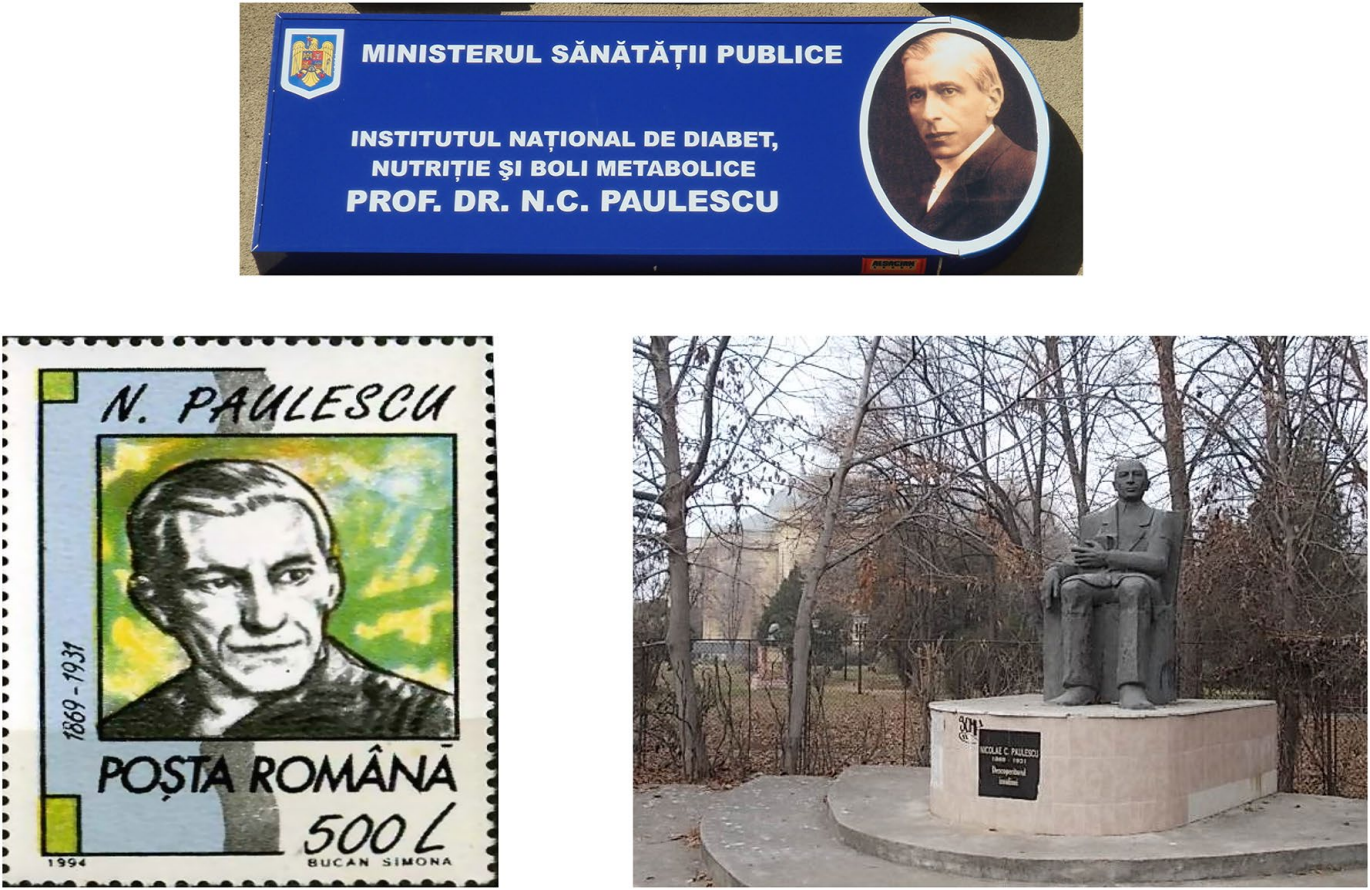
In 1993, a postmark was issued to honor Paulescu. In March of the same year, the Bucharest Institute of Diabetes, Nutrition and Metabolism adopted the name of N.C. Paulescu. In 2001 a bronze statue of Paulescu was erected near the University Carol Davila in Bucharest (photographs taken by the authors in 2007)

In 2001 on the occasion of the 80th anniversary of the publication of Paulescu's article on the discovery of pancreina, a bronze statue of him was erected near Bucharest University of Medicine and Pharmacy. The President of Romania, Ion Iliescu, and the President of the IDF, Sir George Alberti, presided the ceremony. Alberti wrote about this event in the IDF's bulletin. Some excerpts are reproduced below [19] (Fig. 5):

**Table:** 

"I recently had the enormous honor to unveil a large bronze statue of Nicolae Paulescu in Bucharest together with the President of Romania. The occasion was the 80th anniversary of the publication of Paulescu’ seminal paper of his discovery of insulin."
"(…) He (Paulescu) was the first to discover the actions of what was later called insulin and clearly demonstrated that it was a hormone with actions on all aspects of metabolism."
"(…) My own view is that Paulescu’s observations were fundamental to our understanding of insulin, but the Canadians were the first to treat patients successfully. Sufficient credit was not given to the outstanding work of Paulescu."
George Alberti, 2001 [19]

In 2002, the Romanian Academy, the European Association for the Study of Diabetes (EASD) and the International Diabetes Federation (IDF) agreed to organize a series of academic events to pay homage to NC Paulescu:

### A-International Research Award “Nicolae Paulescu”

It was agreed to give an award named after Paulescu, for original investigation of excellence related to the antidiabetic hormone, on competitive and international bases. For this purpose, an evaluation committee was appointed, formed by Robert Heine (The Netherlands), Alberto de Leiva (Spain) and Zvi Laron (Israel) as president [1; pp. 601–603].

Four international experts presented their candidacy. The Commission unanimously decided to award the prize to Geremia Bolli, Professor at the University of Perugia. In accordance with the premises of the call, the awarded researcher would give the “NC Paulescu Memorial Lecture” on August 27, 2003, as part of an academic act to be held at the Hôtel Dieu (Paris), where a commemorative plaque and sculptures of N.C. Paulescu and his mentor, E. Lancereaux, would be installed [20].

## Nicolae Paulescu denounced for antisemitism (Judeophobia)

**Fig. 6:**
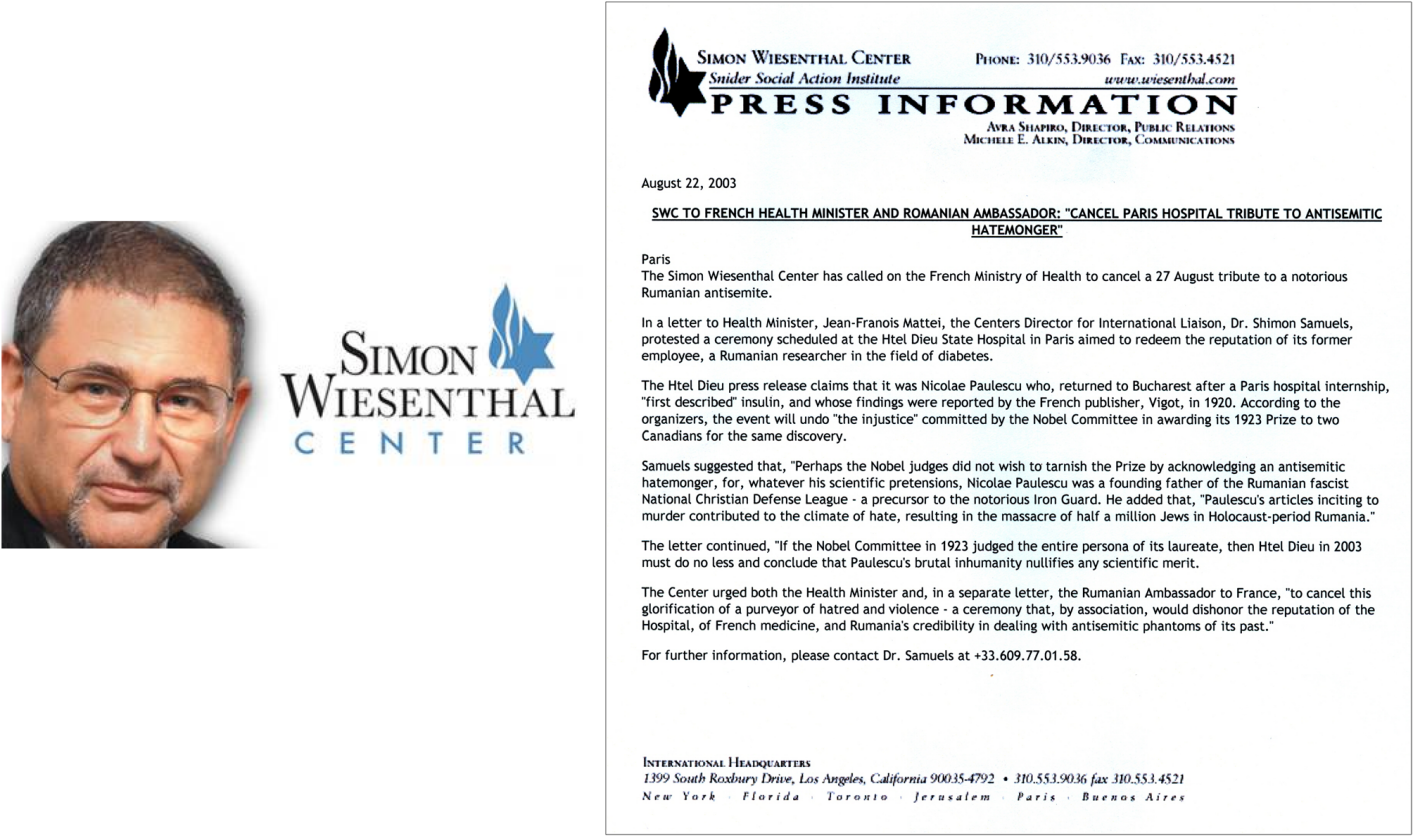
Left: Portrait of Dr. Shimon Samuels, director of Internal Affairs, European Center of the Wiesenthal Foundation (SWC) in Paris. Right: News release from the European Center of SWC to the French Minister of Health, Jean-François Mattei and Romanian Ambassador, regarding “the Paulescu case” (August 22, 2003). Public domain

These celebrations were canceled as a consequence of the reception by the French Minister of Health, François Mattei, and by the Romanian Embassy in Paris of a complaint of antisemitism against the Romanian researcher made by *Dr. Shimon Samuels*, director of Internal Affairs, European Center of the Wiesenthal Foundation in Paris, on August 22, 2003 (Fig. 6) [21]. The Center urged both the Health Minister and the Romanian Ambassador “to cancel this glorification of a purveyor of hatred and violence, a ceremony that, by association, would dishonor the reputation of the Hospital, of French medicine, and Romania’s credibility in dealing with antisemitic phantoms of its past” (Fig. 6) [21].

**Fig. 7:**
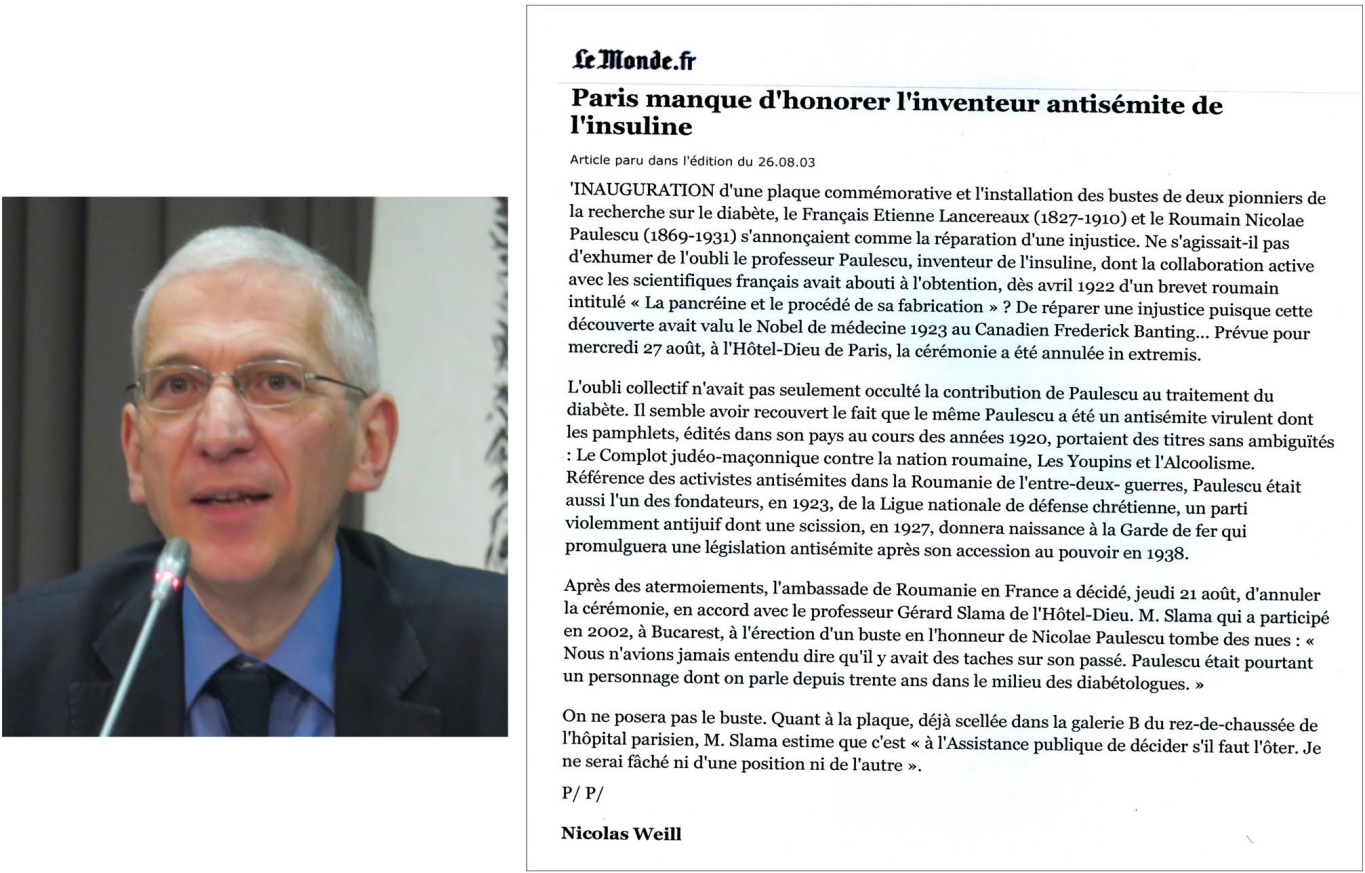
Editorial by N. Weill in le Monde, reporting on the cancellation of the homage in Paris to the “antisemitic inventor of insulin” (08–26-2003). Public domain

On August 26, 2003, *Nicolas Weill*, journalist for *Le Monde* since 1995, expert on the Middle East, published an editorial confirming the claim filed four days earlier by the SCW. Weill reported that the ceremony called to honor Etienne Lancereaux and Nicolae Paulescu had been canceled in extremis. The journalist denounced Paulescu's antisemitism manifested in Judeophobic pamphlets and his role as co-founder in 1923 of the “Ligue Nationale de Défense Chrétienne”*,* a violently anti-Jewish party (Fig. 7) [22].

## Opinions and reactions of various organizations to the denunciation of Paulescu for antisemitism and the cancellation of the scientific tribute to him

*Nicolae Cajal* (1919–2004), an internationally renown scientist born into a Jewish family, director of the Department of Virology at the University of Bucharest, expert of the World Health Organization (WHO), member of the Royal Society of Medicine (London), president of the European Association for the Study of Poliomyelitis, the Department of Medical Sciences of the Romanian Academy, and president of the Federation of Jewish Communities in Romania from 1994 until his death [23], called a press conference on August 31, 2003, in which he defended the scientific merits of Paulescu, and insisted on the need to establish a relevant distinction between his academic contributions and his antisemitic views. At the end of the statement, Cajal affirmed that no one can ignore the scientific merits of Paulescu for his contribution to the benefit of the health of citizens [24] (Supplementary Fig. 3).

On September 29, 2003, *Eugen Simion*, president of the Romanian Academy, and vice president, *Maya Simosnescu*, expressed their disagreement about the cancellation of the act of homage to Paulescu in a letter addressed to Pierre Lefèbvre, President of IDF. In a paragraph they insisted on the essential aspects of the press statement of the Jewish Community of Romania of August 31, 2003, and their solidarity with the statements of Nicolae Cajal (Supplementary Fig. 4).

**Table:** 

“Almost one century after these events we wonder if it is fair to judge the man’s scientific values according to his personal options. They are distinct problems that should be considered as such. We cannot judge the work of the great prose writer Céline according to his political ideas. We cannot take Ernst Jünger out of the twentieth century literature only because he was an officer of the German army. This dissociation is absolutely necessary in science too, or better said**,** specially in science. What is more important in a scientific life: the fact that he discovered insulin an in this way has been saving millions of lives or that in some circumstance of his biography he published a few (true, regrettable) articles with nationalistic tendencies?”
Eugen Simion and Maya Simionescu, 2003 [1: p. 612]

Eugen Simion (1933–2022), literary critic and historian, was Professor of the Department of History of Roman Literature, University of Bucharest and of Romanian language at the University of La Sorbonne (Paris-IV). In 2006 he assumed the general direction of the Institute of History and Literature “G. Calinescu” in Bucharest. He presided over the Romanian Academy between 1998 and 2006 [25].

**Fig. 8:**
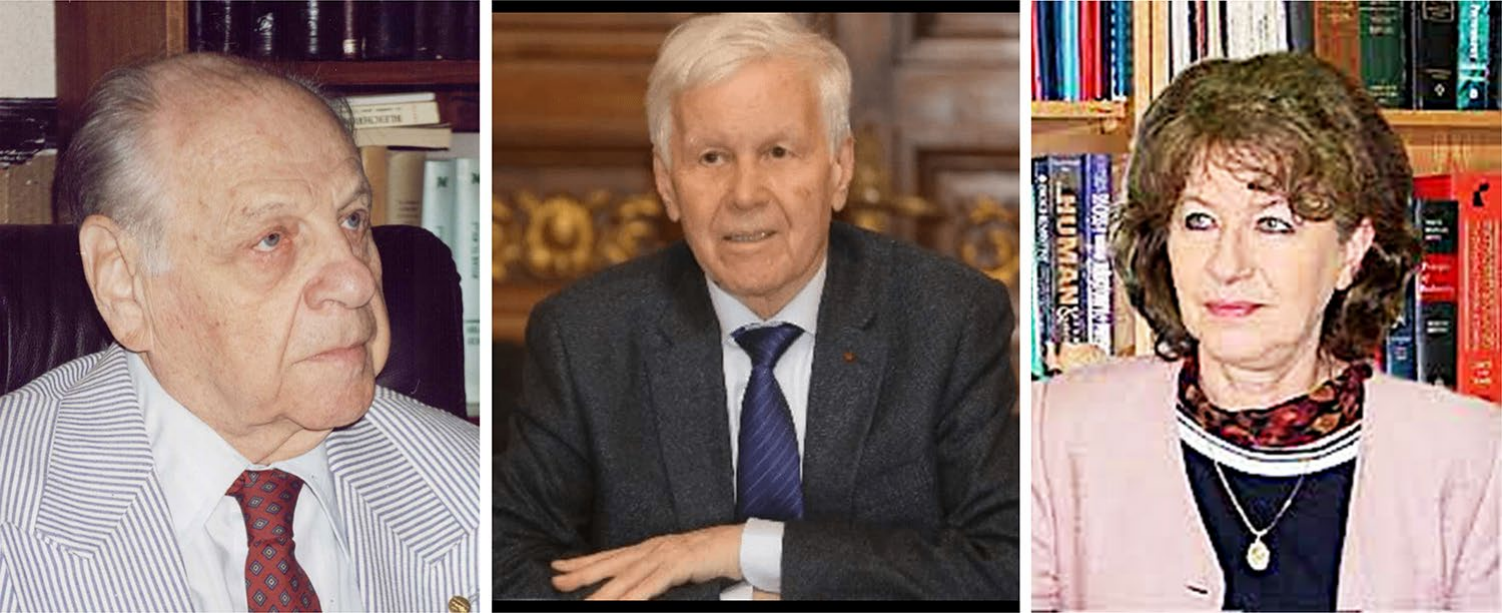
Portrait of Nicolae Cajal. Unknown date and author. Source: Archive/ROMPRES [24] Public domain. Eugene Simion and Maya Simionescu. Source: Romanian Academy. Unknown dates and authors. Public domain

Maya Simionescu received her PhD from the Faculty of Biology, University of Bucharest. After 10-year training under Prof. Georg Emil Palade, Nobel Prize Laureate of Physiology or Medicine in 1974 for his contribution on the functional organization of the cell, worked at Rockefeller University, NY and Yale University, New Haven, CT. After her return to Romania, she cofounded the new Institute of Cellular Biology and Pathology (ICBP). In 2000, ICBP was selected Centre of Excellence of the European Community. At present, she is director of ICBP, member of the Romanian Academy and President of the Section of Biological Sciences and President of the Romanian Society of Cell Biology [26] (Fig. 8).

In October 2003, Prof. *Gérard Slama*, Director of the Diabetes Department, Hôtel Dieu, and Honorary Professor at the Paris V Faculty of Medicine, published a letter in *The Lancet*, that ended with the following words: “It seems to me that the Romanian authorities are extremely reluctant to publicly recognize and condemn Paulescu's past (…). One might ask if, by its conspiracy of silence, Romania has tried to manipulate international public opinion by hiding its antisemitic past behind a public veil”.

Two months later, *George Alberti* and *Pierre Lefèbvre*, President and past President of IDF, wrote a letter to *The Lancet*, referring to Slama's earlier writing, stating that: “The IDF is now collecting the appropriate writings of Paulescu. These will be scrutinized by an independent committee. The IDF does not wish to mix science and politics. But more information is needed before we can internationally laud an individual who has undoubtedly made a major scientific contribution but who may have espoused a morally unacceptable position later in life.” [27, 28] (Supplementary Fig. 5).

## Symposium in Delphi, 2005, on the occasion of the 41st Annual EASD meeting. “Who discovered Insulin?

On September 8, 2005, Christos S. Bartsocas, Spyros G. Marketos, KGMM Alberti, Jørn Nerup, Joh Alivisatos, Stefano Geroulanos and Sotorius Raptis organized an International Symposium of Experts in Delphi on the occasion of the celebration in Athens of the 41st Congress EASD Annual, entitled “Who discovered insulin?” (Supplementary Fig. 6).

The different keynote lectures by Thorstent Deckert (Denmark), Alberto de Leiva-Hidalgo (Spain), Constatin Ionescu-Tirgoviste (Romania), John Dupré (Canada), Jay Skyler (USA) and Paolo Pozzilli (Italy) reviewed the most significant progress in the history of the discovery of the antidiabetic hormone and confirmed that international organizations had ignored the scientific contributions of NC Paulescu for many years.

In the last speech, *Zvi Laron* denounced Paulescu's antisemitism and explained the terrible consequences of the Romanian Holocaust. The magnitude of these revelations led to widespread confusion, and the symposium's organizing committee decided to cancel the debate, the voting and the drafting of a final document, provided for in the program.

In the same year, 2005, the Executive Committee of the IDF declared that the Federation did not want to associate its name with that of NC Paulescu and for this reason the Paulescu Conference would not be convened in the official programs of future IDF world congresses [1; pp. 616–617]. Three years later, Z. Laron wrote an article about Paulescu, that concluded with the following remarks [29]:

**Table:** 

"Paulescu may be acknowledged for his scientific work, but unquestionably should not be feted and honored. Quite the contrary -he should be unequivocally censured for his contribution to the dark pages of Romanian history"	

## Antisemitism in Romania

Hostility toward Jews has a long tradition in Europe, that's why it's sometimes called "the longest hatred" [30]. Philosopher Hannah Arendt, author of *The Origins of Totalitarianism*, described Romania as "the most antisemitic country in pre-war Europe" [31]. In the late nineteenth and early twentieth centuries, renowned Romanian intellectuals, among whom Nicolae Paulescu, helped to spread antisemitist propaganda. To try to understand why the Romanian cultural and scientific elite espoused antisemitism, it is necessary to give some background information on the historical context of judeophobia.

William I. Brustein, a widely-published scholar in the areas of political extremism and ethnic, religious and racial prejudice, identifies four historical forms of antisemitism in Europe: religious, economic, socio-political, and racial [32]. The roots of Romanian antisemitism lie in a combination of these four streams and are intertwined with the political upheavals of the process of Romanian nation-building.

Present-day Romania includes four major historical provinces: Transylvania, Wallachia, Moldavia, and Dobroudja. In 1859, the principalities of Wallachia and Moldavia, which were under Ottoman rule, united following the election of Alexandru Ioan Cuza as prince of the two. In the Russo-Turkish War (1876–1877), Wallachia and Moldova fought on the Russian side. In the aftermath of the Russian victory, by virtue of the Treaty of Berlin (1878), Romania was recognized as a sovereign independent state by the European Great Powers, provided that it accepted some territorial changes (Bessarabia—the eastern part of Moldavia—was given to Imperial Russia, while Romania incorporated a part of the region of Dobroudja), and that Romania also granted full citizenship rights to the Jews, who composed 4.3 per cent of the Romanian population [33, 34]. The Romanian Government fought to keep this provision out of the Treaty and the "Jewish question" became an integral part of public debate.

Prominent politicians argued that the integration of Jews in Romanian society would jeopardize the State’s Romanian national character. The process of nation building was accompanied by the development of a populist, xenophobic and antisemitic discourse [35]. Jews were also seen as a threat to the emerging Romanian middle class, since most Jews made their living in crafts and commerce, as well as in the liberal professions. Well-known intellectuals like Mihai Eminescu, Alexandru Xenopol, Nicolae Iorga, Vasile Conta, amongst many others, spread antisemitism warnings about the threat that Jews represented to Romanian culture [36]. Important members of the Romanian Orthodox Church also contributed to the consolidation of popular antisemitism.

The Jews of Romania were the last in Europe to obtain citizenship, as late as 1919. The antisemitic discourse was further inflamed by theories of race and eugenics.

## Race and Eugenics in interwar Romania

*Social Darwinism*, a theory popular in the late nineteenth and early twentieth centuries, that misapplied Darwin’s natural selection to human groups, held that the human existence was ruled by the “survival of the fittest” [37]. Social Darwinism was used to develop a scientific discourse about "racial" differences and to justify racist policies. The new concept of nation integrated geography, historical destiny, and biological terms. Anthropometric parameters and the composition of the blood became important somatological characteristics, inseparable from racial identity [38].

Craniometry, physiognomy, and phrenology, that were concerned with measuring the human body, were used to identify "racial" differences. For instance, Victor Papilian (1888–1956), Romanian anatomist, writer and university professor, used craniometry to differentiate between populations from Transylvania (where there were people of Hungarian origin) and Romanians from the Old Kingdom [39].

Eugenics became a common practice for state interventions to prevent defective individuals from procreating. Sexual sterilization was justified to improve the biological qualities of future generations [40].

Gheorghe Banu (1889–1957), a Romanian hygienist and Health Minister in the Octavian Goga cabinet (1937–1938), declared abortion, segregation, and prophylactic sterilization, as effective eugenic measures. The sterilization of imbeciles, idiots, epileptics, criminals, and psychotics, as well as individuals suffering from syphilis, tuberculosis and leprosy allowed the conservation and improvement of the race [41, 42].

## Antisemitism in the interwar period in Romania

After World War I, pursuant to the Peace Treaty of Paris (1919), Romania acquired the provinces of Bessarabia (from Russia), Transylvania and the Banat (from the Hungarian half of the Austro-Hungarian Empire) and Bucovina (from the Austrian half of Austria-Hungary) and formed Greater Romania (*România Mare*). Romania's territory and population doubled. Jews, particularly in the annexed territories, were perceived as foreign hostiles and "were constantly accused of having pro-Hungarian or “Bolshevik” inclinations" [43].

Antisemitism was rampant throughout all layers of Romanian society. Jews were considered a triple threat in Romania: religious, ethnic and political. The Romanian press called the Jews *usurers, purveyors of adulterated alcohol, spies and blood suckers*. Instead of the neutral word “evreu” (Jew), the derogatory *jidan* gained currency [44].

**Fig. 9:**
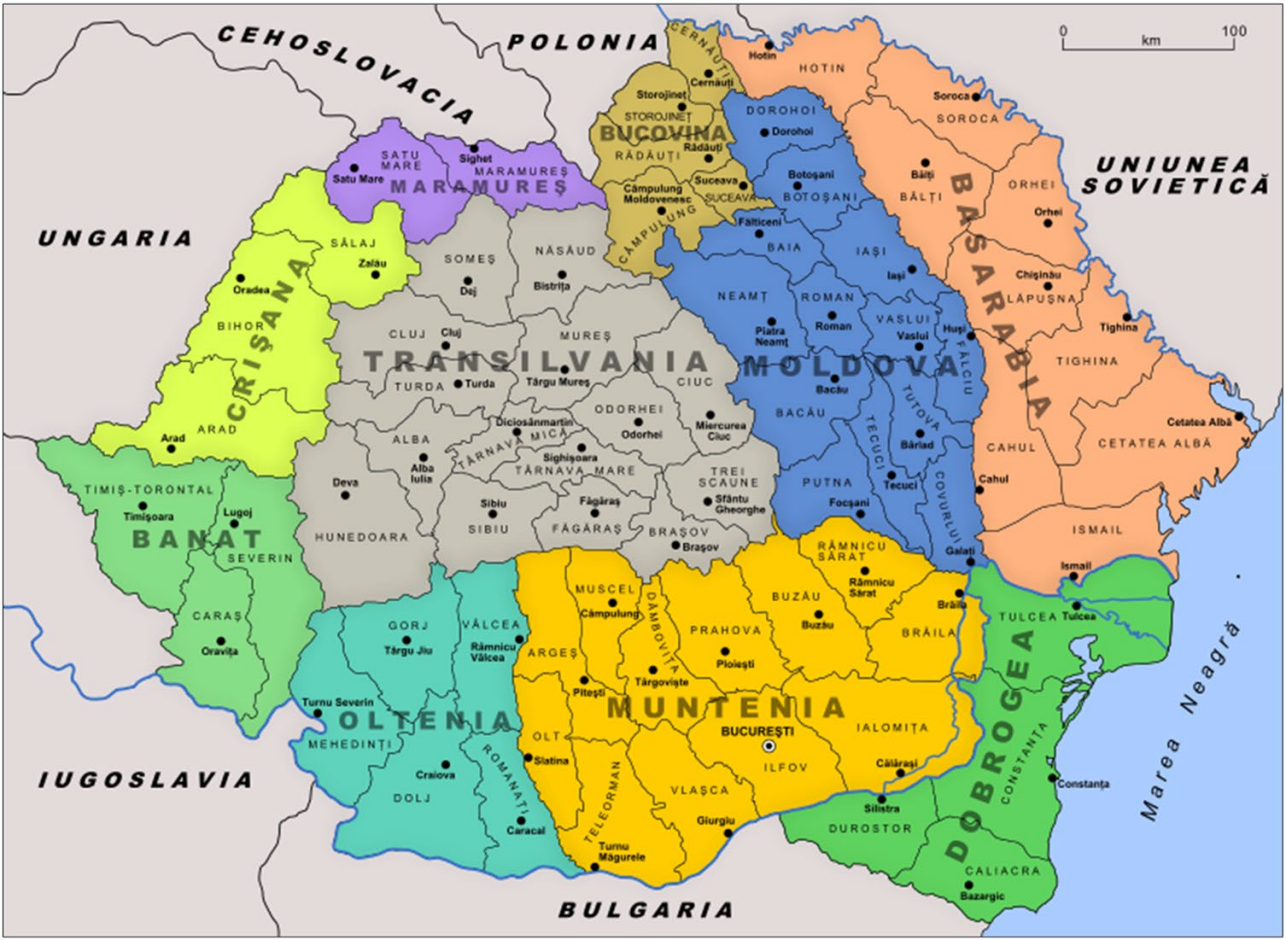
Administrative map of Romania in 1930. (Public domain)

*Alexandru C. Cuza* (1857–1947), Professor of Law at Iaşi University, founded the “Liga Antisemita Universala” in 1895. In 1910, Cuza and the historian and writer Nicolae Iorga (1871–1940) established the “Nationalistic Democratic Party” as a reaction to the arrival of Jewish refugees fleeing from Russia, mainly to Transylvania, Bessarabia, Northern Bucovina, and part of Dobruja (Fig. 9).

Marius Turda, founder of the International Working Group on the History of Race and Eugenics and Deputy Director of the Centre for Health, Medicine and Society at the School of Arts and Humanities, Oxford Brookes University, pointed out that [45]:

**Table:** 

(…) The Jews became “undesirable” both politically and medically. Degeneration was one of the arguments used most consistently in stigmatizing the Jews and opposing them to the “healthy” Romanians
(…) Romanian doctors thus envisioned a national community based upon the exclusion of those to be “alien”, “hereditary ill”, or “antisocial”
(…) The Romanian national community itself was categorized in according with racial criteria. These criteria included not only ideas of “racial purity” but also biological measures against Jews

The incorporation into Romania of Jews from former Hungarian and Russian territories linked antisemitism to anticommunism. The Romanian Communist Party included among its leaders’ members of ethnic minorities, especially Jews, like Ana Pauker, Marcel Pauker and Alexander Dobrogeanu-Gherea, heads of the communist movement in the interwar period [1: pp. 626–627].

In March 1923, the Romanian Government proclaimed a new constitution that offered the nationality to minority groups, including Jews. A new campaign of protests showed severe hostility against Jews with the complicity of a good number of intellectuals (Vasile Conta, Vasile Alecsandri, Mihai Eminescu and Ioan Slavice, among them) [46].

**Fig. 10:**
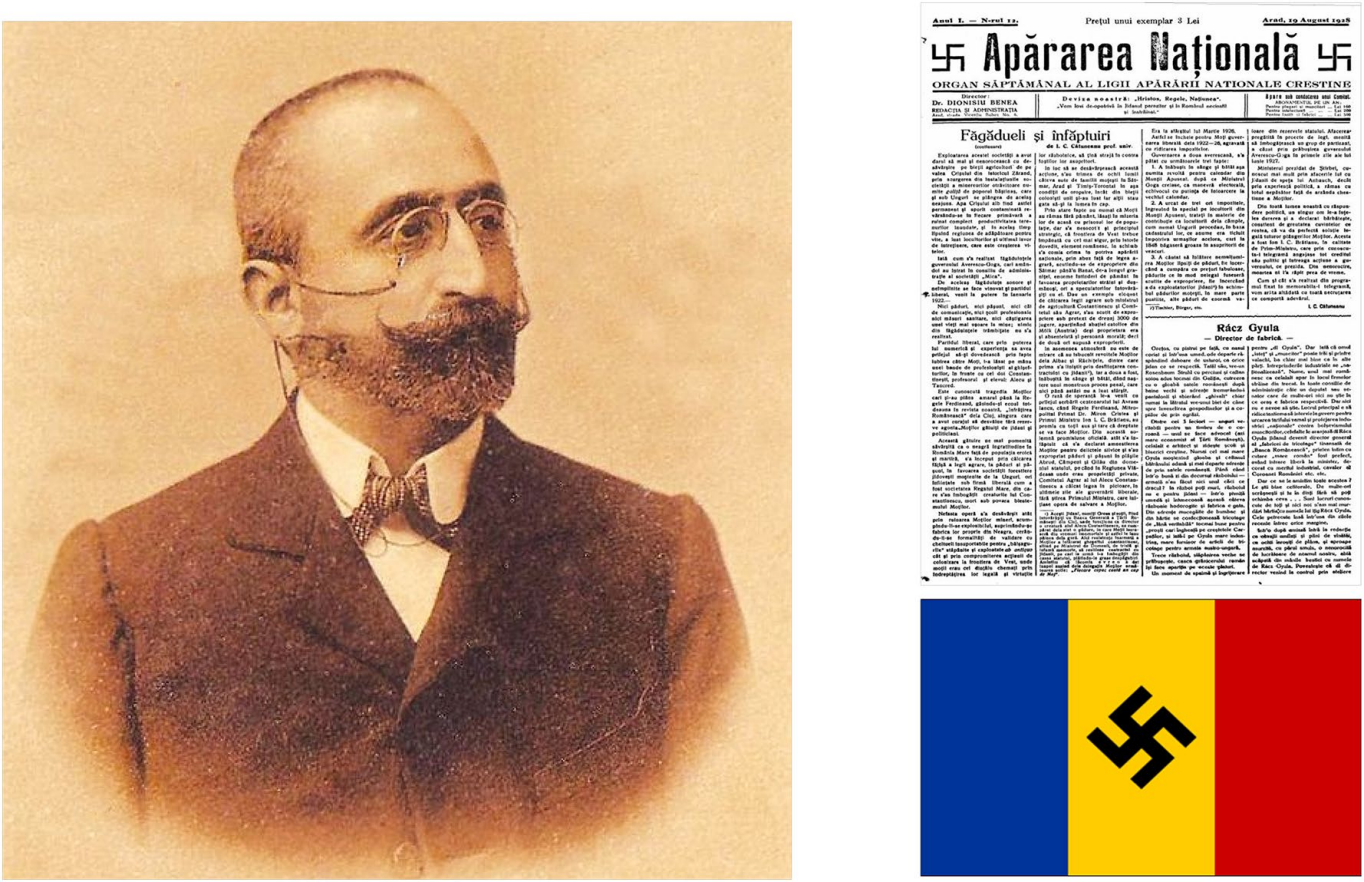
Alexandru C. Cuza (portrait) and other Romanian intellectuals (Paulescu among them) founded the *Liga Apǎrǎri National Creştine* (LANC, League for Christian National Defense). The official Newsletter of the organization was *Apǎrarea Nationalǎ.* Source: Selected records related to A.C. Cuza and the National Christian Party (United States Holocaust Memorial Museum Archives). Public domain

Alexandru Cuza, member of the Parliament, created in 1923 the “Liga Apǎrǎrii National Creştine”*, LANC* (League of National Christian Defense), with the participation of five prominent intellectuals (including Nicolae Paulescu). The LANC had its roots in the Christian National Union (UNC), created in 1922 by Cuza and Paulescu. The main purpose of the UNC was to support Romanian economic, political and cultural interests against the Jews: to deny their emancipation; to expel the Jewish population arrived in Romania after 1914; to apply the *numerus clausus* against Jews in schools, businesses, industries, universities and liberal professions, and banning Jews from electoral lists. The symbol of LANC was the flag of Romania with a svastika at the center. For them, the svastika represented the "sign of the Romanian nation and of its most distant past and symbol of the will to preserve ourselves in the future" (Fig. 10) [1; p. 650–653].

Cuza and Paulescu were in favor of parliamentarianism, while *Corneliu Zelea-Codreanu,* a young law student, leader of the extreme radical orientation of LANC, tried to get the League to organize itself on the model of the fascist and paramilitary formations present in various countries in Europe at the time. The League participated in the general elections of 1926, with the support of Octavian Goga, Minister of the Interior, obtaining 4.76% of the votes. Nicolae Paulescu unsuccessfully aspired to the position of senator in the county of Ilfov (district of Romania in the Muntenia region, which surrounds Bucharest). Due to LANC's radical racism, Nicolae Iorga decided to leave the association with Cuza. Iorga would later accept the granting of Romanian citizenship to all Jews. Cuza maintained his radical antisemitism, although betting on the parliamentary path.

In 1927, Codreanu abandoned LANC and created the fascist antisemitic party, “Legiunea Arhanghelului Mihai” (Legion of the Archangel Michael), that would be later known as “Garda de Fier” (The Iron Guard). Relevant intellectuals joined the movement (Nichifor Crainic, Nae Ionescu, Mircea Eliade, among others); they adopted messianism, the cult of personality, and anti-communism, exalting orthodox Christian values ​​accompanied by a spiritual aura. The legion chose as its main symbol a triple cross, that represented the bars of prison or shield of martyrdom (Cross of the Archangel Saint Michael). Its members wore green shirts (a sign of rejuvenation) and the Roman salute. A.C. Cuza, president of LANC, despite the ideological communion, disagreed with the violent tendency of the “legionnaires”, which generated a distance between both organizations [47].

The legion carried out assassinations of politicians they considered corrupt, including the Premier Ion G. Duca. Codreanu advocated Romania’s adherence to Nazi Germany [48].

William I. Brustein, A. Ronnkvist and Ryan D. King, looking at data from 1899 to 1939, a period of intensified antisemitism in Western European countries, concluded that Romanian antisemitism during this period was characterized by a higher degree of violence than in the other European countries investigated (Great Britain, France, Germany and Italy) [49].

In December 2003 we consulted the Simon Wiesenthal Center. Then we continued the investigations on “the Paulescu case” by visiting institutions and archives and interviewing relevant professionals in Bucharest who knew first-hand about Nicolae Paulescu's socio-political activities. A summary of these activities can be found in the Supplementary Material (3): “Consultation of the Simon Wiesenthal Center and results of our visit to Bucharest in 2007”.

## N.C. Paulescu political activism

**Fig. 11:**
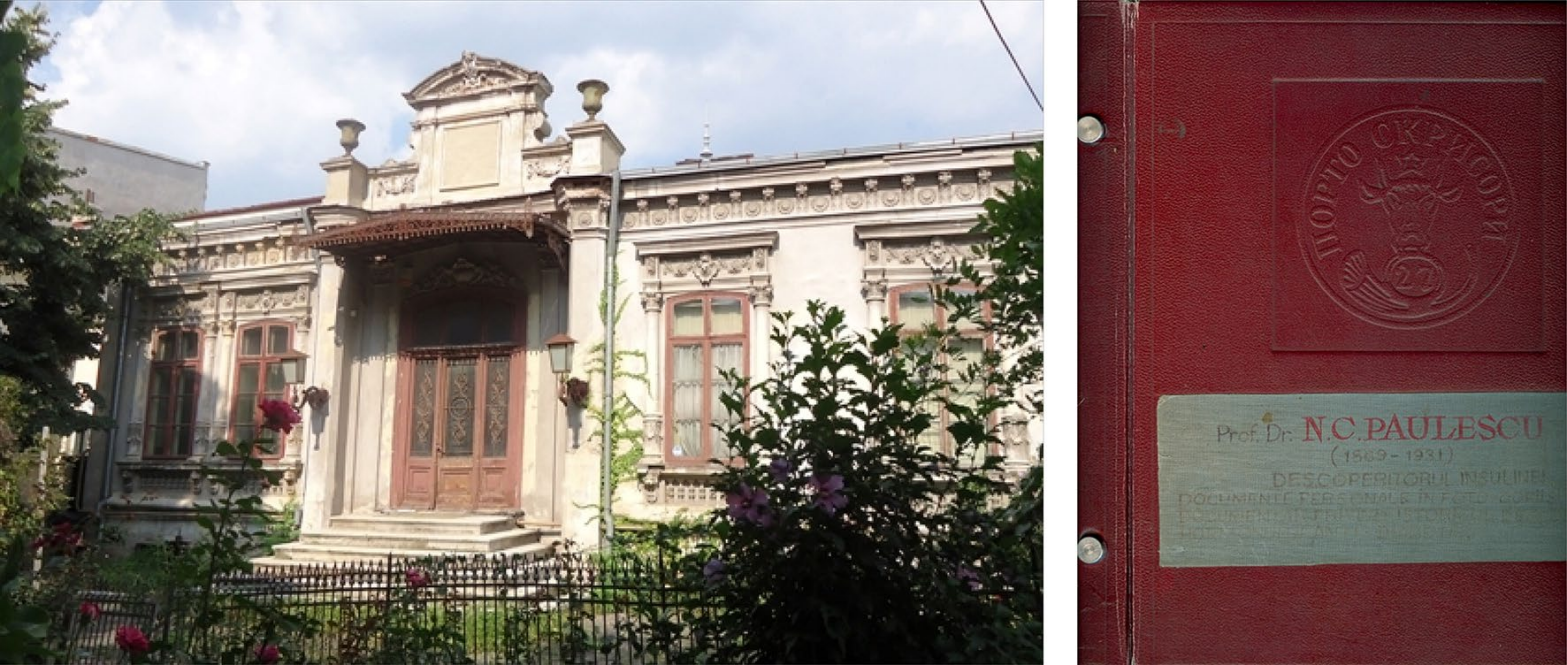
NC Paulescu House-Museum (Calonfirescu, 15 St, intersection with Hristo Bolev). Cover of Paulescu's private album with documents and images. Courtesy of Dan Angelescu. Photographs taken by the authors, October 2007

**Fig. 12:**
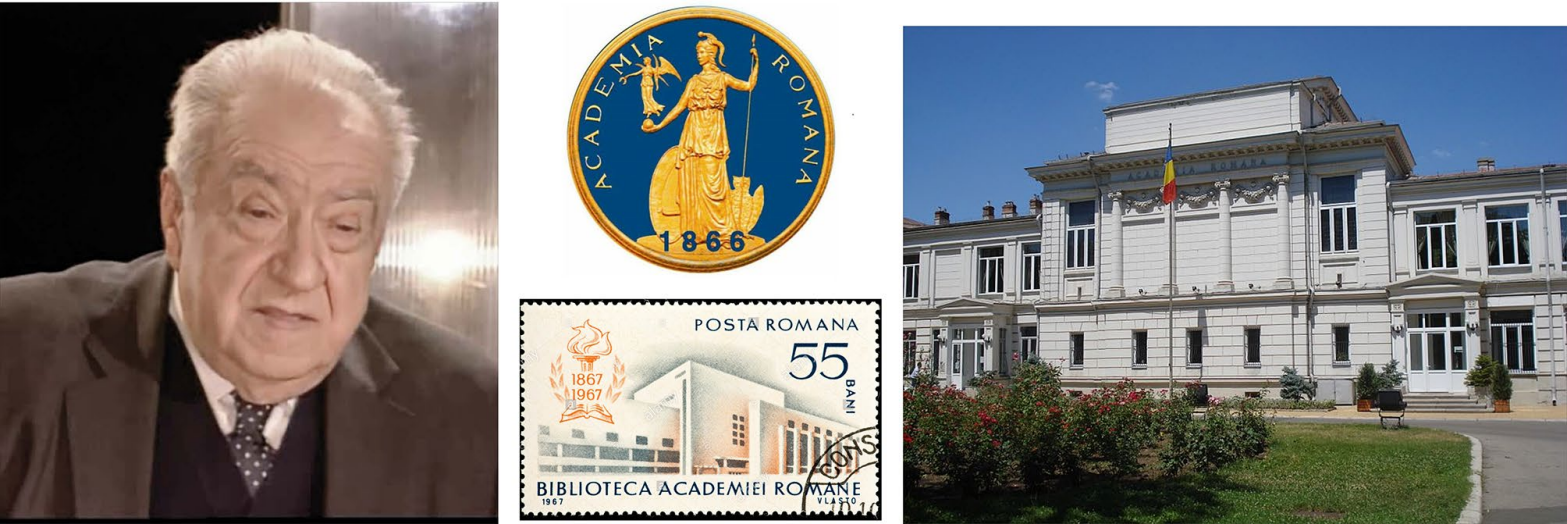
Portrait of Gheorghe Brǎtescu. Unknown author and date. The building of the Romanian Academy, the seal and a postmark. Public domain

We interviewed Professor *Gheorghe Brǎtescu* in the living room of Paulescu's House-Museum (Fig. 11). Brǎtescu (1922–2017), a physician affiliated with the Romanian Communist Party in his youth, abandoned professional politics in 1956, to dedicate himself entirely to research in the History of Medicine. He was Vice-President of the Romanian Society for the History of Medicine, President of its Bucharest committee (1977–1986), member of the Romanian Academy and Honorary member of the Swedish Society for the History of Medicine, among other honors (Fig. 12) [50–52].

During the interview, Brǎtescu was accompanied by his wife, Tatiana Parker, daughter of the communist political leader Ana Parker. Brǎtescu described to us in detail what he called the "drama of Paulescu", a great scientist but unfortunately politically deviant on the extreme right and a radical fanatic antisemite [53].

**Fig. 13:**
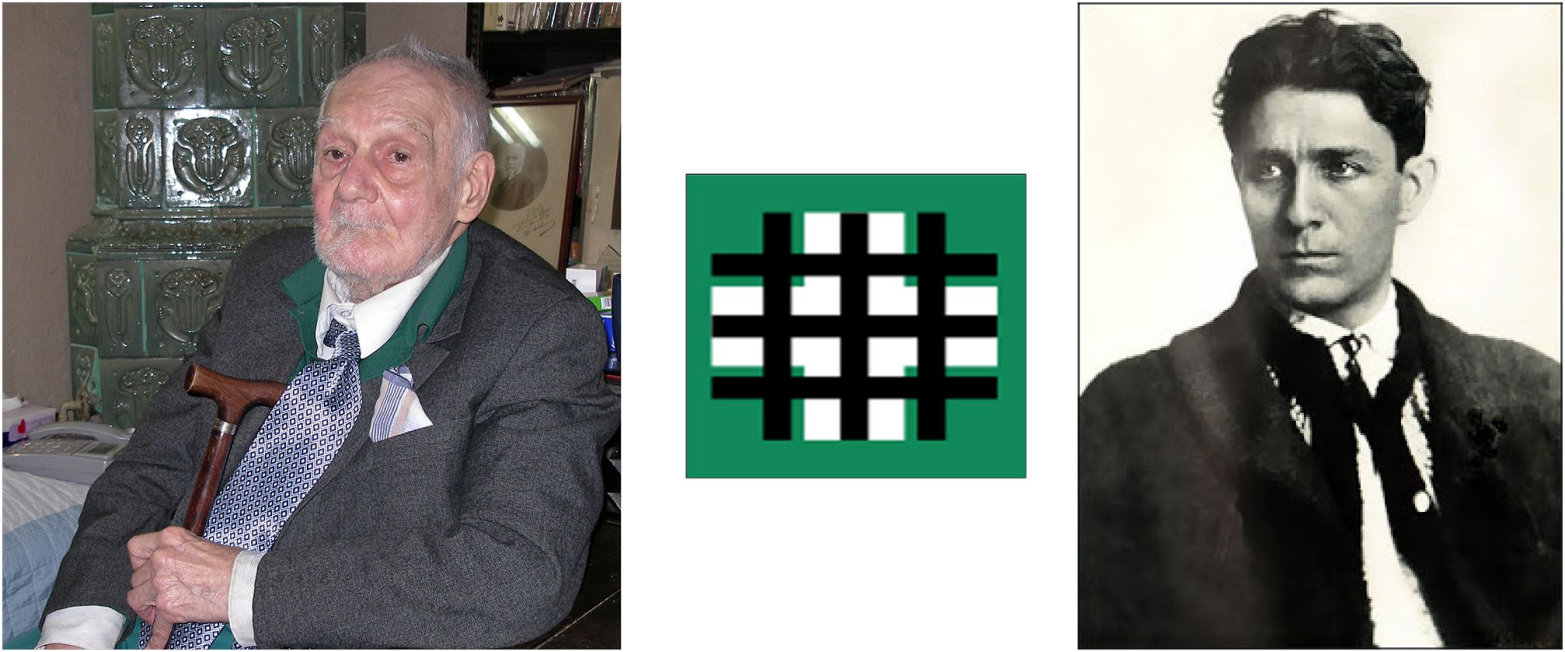
Left: Portrait of Serban Milcoveanu (1913–2009), old student of Paulescu (1930–1931) and President of the Union of Christian Students of Romania (1937–1940). (Photograph taken by the authors in October 2007). Right: Portrait of Corneliu Zelea. Codreanu (unknown date and author). The main symbol of the Legionary movement was the triple cross or prison bars. Public domain

On October 20, 2007, we conducted an interview with Dr. *Serban Milcoveanu* (1913–2009) at his home. Milcoveanu, a disciple of Paulescu (1930–1931), was president of the National Union of Christian Students of Romania (1937–1940) and felt a deep admiration for Paulescu, with whom he shared political and religious ideas (Fig. 13).

In the interview, Milcoveanu confirmed to us that LANC, antisemitic political party, was chaired by Prof. Alexandru C. Cuza (Law Faculty, University of Iaşi). Five other university professors, important figures of the Romanian intellectual spheres, were co-founders: Ion Catuneanu (Roman Law, Cluj-Napoca University), Ion Gavanescu (Pedagogy, Iaşi University), Nicolae Paulescu (Physiology, University of Bucharest), Constantin Tomescu (Theology, Chisina University) and Corneliu Sumuleanu (Chemistry, Iaşi University) [14; pp. 651–652].

Milcoveanu, devotee of Cornelio Z. Codreanu, was active in the legionary movement until Codreanu was assassinated in 1938 [54].

## Main ideological documents written by NC Paulescu.

The extensive list of documents and pamphlets written by NC Paulescu, available as Supplementary Material (4), has allowed us to figure out the religious and sociopolitical ideology of the Romanian scientist, which we present in summary form:

1. Antidarwinism

Paulescu was opposed to both spontaneous generation and darwinism. He did not admit evolution. He believed that the species of living beings, both plants and animals, were invariable or fixed [55, 56].

2. Orthodox Christianity

Paulescu was a creationist. In his writings, conferences and lectures he tried to rationalize the figure of the creator in biological terms, as well as the existence of the soul and the errors of materialism.

**Table:** 

(…) God is the initial cause and the final scope of everything that exists. True science can only come to decipher the desires and reasons for the divinity of Jesus Christ. (…) The scientist not only says: **Credo in Deum**. It has to affirm strongly **Scio deum esse**	

**Fig. 14:**
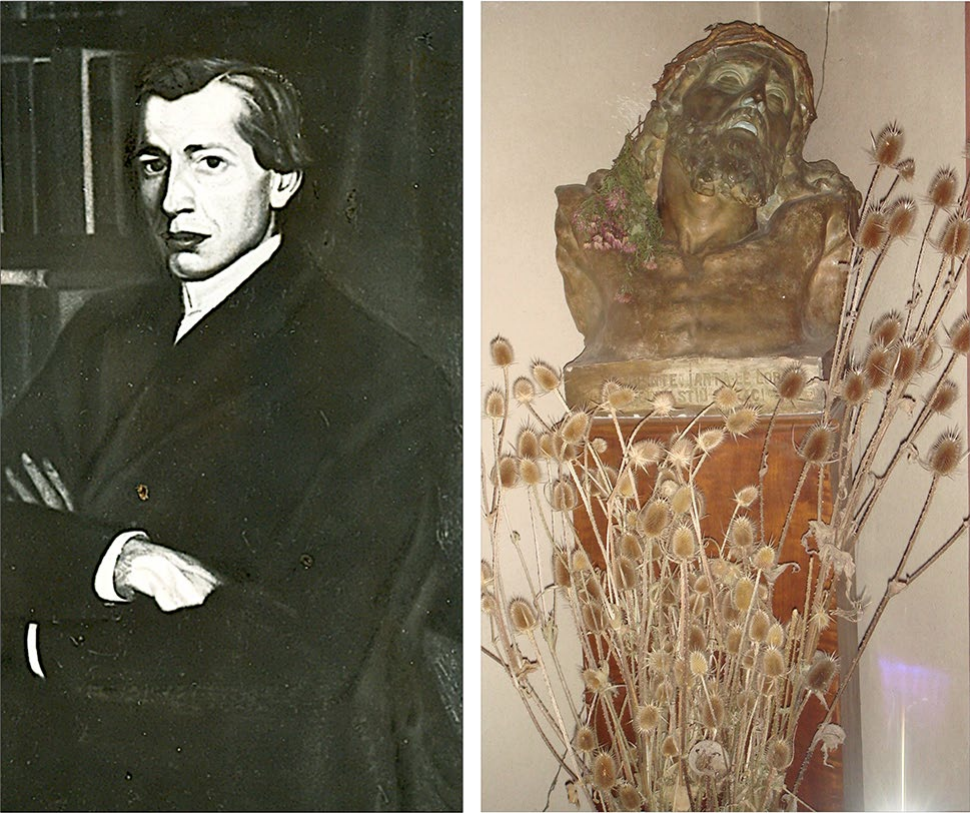
Left: Portrait of Paulescu in 1906, painted in oil by C. Artachino. Right: Sculpture of Jesus Christ, the work of Dimitri Paciurea, dated 1907. Photographs taken by the authors in the hall and bedroom, respectively, of Paulescu's home in October 2007

The antisemitism professed by Paulescu was fruit of his Christian-orthodox radicalism. He was deeply devoted to his love for Jesus Christ. Nichifor Crainic (1889–1972), theologian and member of the Romanian Academy, popularized at numerous conferences and events organized by the Institute for Christian Studies, the figure of Paulescu as the Christian-nationalist prophet of Romania. The physician Vasile Trifu wrote in 1930 that Professor Paulescu had clarified the resurrection of Christ from a scientific point of view. This explains the sculpture we saw in Paulescu's bedroom, a bust of Jesus crowned with thorns, the work of the sculptor Dimitrie Paciurea, probably the best representative of Romania's sculptural avant-garde. Paulescu maintained personal relationships with Romanian contemporary intellectuals and artists. Among the art objects present in Paulescu's house, we were particularly struck by Paulescu's oil portrait painted by Constantin Artachino (1870–1954), a founding member of the Society of Young Romanian Artists, with N. Vermont, S Popescu and S. Luchian, among others. Artachino taught at the Bucharest School of Fine Arts between 1920 and 1935 (Fig. 14) [1, 57].

3. Religious antisemitism

Paulescu located the origins of "Jewish treachery" in the *Talmud* (the central text of Jewish theology). Liberalism, socialism and Marxism had their origin in the *Talmud*. Paulescu's antisemitism was, for the most part, a consequence of his Orthodox-Christian radicalism. Deeply devout, he had come to believe in the myth of Jewish ritual murder recounted in the “Book of the Kahal” [58].

Paulescu compared the Koran and Islamic laws with Jewish laws, insisting that the Talmud's teachings did not include moral obligations toward gentiles. He believed that the activities of the Kahal and Freemasonry were intertwined with Judaism in its bid for world domination and the destruction of Christian civilization. He considered the Kahal as the factor of corruption that threatened the state [59].

Paulescu believed that Freemasonry was the primary means used for the destruction of Christian civilization by removing access to trade and industry to all individuals of other religions and by concentrating all capital and real estate in their hands [60].

According to his interpretation of the Apocalypse: “The world is divided into Christian people (“divine Christianity”) and Luciferian people (“demonic Judaism”). He relied on a distorted version of the New Testament to decipher the divine will for the extermination of the Jews. In this writing Paulescu's vision of the Christian church is that of a universal antisemitic organization [61].

Paulescu's ideas of religious antisemitism were widely shared by Alexandru Cuza and set forth in articles in the “Apǎrarea Naționalǎ” (National Defense) magazine on "alleged real cases" of ritual murders of Christian children at the hands of Jews [1; pp. 658–660).

“Can we perhaps exterminate them in the ways bedbugs are killed? That would be the simplest, easiest and fastest way to get rid of them", he wrote. But added: "but no! we mustn't even think of that, because we're Christians" [62].

4. Ultra-nationalist political antisemitism

According to Francisco Veiga, professor at the Department of Contemporary History at the Autonomous University of Barcelona (UAB), Paulescu was influenced by Gougenot des Mousseaux (1805–1876), a French antisemitic journalist and writer [63].

Paulescu thought that Jews wanted to obtain world power, and that the Talmud instructed the Jews to obtain this objective and accused the Jews of using alcohol to exterminate the Romanian nation. In his own words [64]:

"(…) Wherever they go, the Jews begin to create clandestine taverns, where they accustom the indigenous people to drink: after having stunned them, they loot all their property and end up killing them.

(…) With the invasion of the hordes of Galiția, this horrific social scourge arose in our country that today is about to exterminate the people.

(…) Alcohol represents the most feared weapon of conquest of this infamous race that wants to exterminate us to take over our country".

5. Racial antisemitism

Paulescu believed in the theory of the "Jewish degeneration as a race" and connected it with crime and the affinity for political movements such as anarchism, socialism and communism. Paulescu based these ideas on the book by the criminologist and professor of Psychiatry in Turin Cesare Lombroso, “L’Uomo delinquente” [65] and the atlas published in France*,* “L'Homme criminel” [66].

Paulescu wrote the following:

**Table:** 

“The Jews have an asymmetrical head, being microcephalic or macrocephalic, oblique and narrow forehead, similar to monkeys, aquiline nose like birds of prey and crossed eyes, drooping ears, long and misshapen, similar to the ape-like type (…). Although degeneration is hereditary, the Talmud imposes on the Jews a new cause of degeneration: marriage between blood relatives. The two causes combined worsen and perpetuate the degeneration of the Jewish race” [67]	

## NC Paulescu: Final Report of the International Commission on the Holocaust in Romania

The international Commission on the Holocaust in Romania was established on October 22, 2003. Elie Wiesel, honorary member of the Romanian Academy and Nobel Peace Award (1986) accepted to be the chairman of the Commission by invitation of Ion Ilescu, President of Romania. The main objective of the Commission was to investigate the truth about the Holocaust in Romania and their preceding events. International experts in history, humanities and social sciences, survivors of the Holocaust, members of international organizations and delegates of the Romanian Presidency, participated in three meetings (Washington DC, May 16–22, 2004; Jerusalem, September 6–9, 2004; Bucharest, November 8–13, 2004). On November 11, 2004, the final report was presented to the President of Romania.

The essential paragraph of the document addressing the obsessive antisemitism of Nicolae Constantin Paulescu can be found in page 35. The most relevant excerpts are reproduced below [68]:

**Table:** 

(…) Paulescu found the origins of Jews perfidy in the Talmud, which he determined was a tool for the extermination of other nations, and the kehillah, which he argued secretly plotted the disasters that afflicted the rest of mankind. While he could not have anticipated the Nazi death camps, Paulescu condemnations of the Jews was so total that he even went so far as to raise the possibility of exterminating the infesting evil parasites in the way bedbugs are killed
(…) Interestingly, not only was Cuza influenced by Paulescu, but the young Corneliu Zelea Codreanu, future founder of the Iron Guard, specifically acknowledged the powerful impact of Paulescu’s ideas on his development

## Final remarks

The Judeophobic content and outbursts of these texts are surprising. It is incompatible to reconcile the scientific rigor and honesty of Nicolae Paulescu's biological experiments with the acceptance of such far-fetched and confused ideas. His Judeophobic obsession distorts the rationality of his thought and discourse.

Paulescu died in 1931, years before the Iron Guard seized power in Romania for a short while in 1940, period during which this fascist movement carried out violent antisemitic attacks against Romanian Jews, but Paulescu did play a pivotal role in the spread of antisemitism and was revered by members of far-right political groups.

However, let's not fall into what historians’ call "presentism", and judge the past with present criteria. James H. Sweet, president of the American Historical Association until January 2023, wrote: "Doing history with integrity requires us to interpret elements of the past not through the optics of the present but within the worlds of our historical actors" [69]. Therefore, a biography of Paulescu should incorporate the lights and darkness of his figure, and, while tracing the influence he exerted on antisemitic organizations and condemn his judeophobia, place him in his historical context, not to exempt him from his responsibility in spreading hateful ideas about Jews, but to better understand the time and place he lived in.

Antisemitism, though, was not confined to Romania. The years 1899–1939 are widely regarded as a high point in antisemitism in Western societies [49]. During the eighteenth and early nineteenth century, under the influence of the liberal ideas of the Age of Enlightenment and the French Revolution, Jews in many European states achieved legal equality, were allowed to leave the ghettos, participate in the trades and professions from which they had been excluded, and vote. Modernization theories of antisemitism argue that Jewish upward social mobility caused friction with non-Jews and heightened antisemitic sentiments [70]. A seminal example of antisemitism in nineteenth century Europe is the Dreyfuss Affair, that involved a French captain of Jewish-Alsatian descent, Alfred Dreyfus, who was falsely convicted of treason for allegedly selling military secrets to the Germans in 1894. The trial sparked a wave of antisemitic riots and became an international *cause célèbre*.

Another example that reveals how deep-rooted antisemitism was in the early twentieth century is the text *The Protocols of the Elders of Zion*, a forged document that claimed to reveal a Jewish plot for global domination, first published in Russia in 1903, translated into many languages and circulated widely internationally [71].

Therefore, antisemitism in the early twentieth century permeated almost every social strata.

### Supplementary Information

Below is the link to the electronic supplementary material.

Supplementary file1 (DOCX 37 KB)

Supplementary file2 (PPTX 53111 KB)
