# Supplementary material for: On the occasion of the centennial of the Nobel Prize in Physiology or Medicine, 1923: Nicolae C. Paulescu—between scientific creativity and political fanatism
Source: Acta Diabetol. 2023 Jul 5;60(11):1513–30. doi: 10.1007/s00592-023-02136-6 (PMC10520201; doi:10.1007/s00592-023-02136-6)
Supplement: Supplementary file 1 — Supplementary file1 (DOCX 37 KB) [file 592_2023_2136_MOESM1_ESM.docx]

**SUPPLEMENTARY MATERIAL**

**Supplementary Material (1)**

**Additional information on the scientific biography of FG Young**

Frank George Young (1908-1988)’s work about diabetes began under J.J.R. Macleod at Aberdeen, and later, under C.H.Best at the University of Toronto. In 1935 he returned to University College, London. Afterwards, he was appointed to the Scientific Staff of the Medical Research Council’s National Institute for Medical Research, under the Directorship of Sir Henry Dale, mentor and friend of Charles H. Best (Dale had proposed Best for the Nobel Award, in 1950). Young was Vice-president of the British Diabetic Association from 1948, President of the EASD (1965-1968) and the IDF (1970-1973).

**Supplementary Material (2)**

**Paragraph of the letter sent by FG Young to I Pavel on June 1971**

Young to Pavel (June 23rd, 1971): "It was my loss that I could not have known personally Prof. N.C. Paulesco. As young research student I spent a period in Aberdeen with the late Prof. J.J.R. Macleod, and in Toronto with Prof. C.H. Best. In Toronto I also came to know Prof. J.B. Collip and Dr. F.G. Banting. I knew Dr. C.H. Best most intimately and am a great admirer of him personally and of his research activities (…). In my capacity of Chairman of the International Committee which has now submitted its report, I attempted to be as objective as possible and to ignore all but evidence published in medical and scientific journals at the time of the discovery of insulin, or shortly afterwards."

**Supplementary Material (3)**

**Consultation of the Simon Wiesenthal Center and results of our visit to Bucharest in 2007.**

We consulted the Simon Wiesenthal Center (SWC). In December 2003, Mrs. Marylin Saqui de Sannes, assistant to Dr. Shimon Samuels, provided us a preliminary list of NC Paulescu's publications, which was expanded with the collaboration of *Radu Ioanid*, director of the US Holocaust Memorial Museum’s International Archival Programs Division. We also received copies of letters sent on August 22, 2003, to the Romanian ambassador in Paris, to Simon Wiesenthal in Vienna, to Robert Marvin Hier, dean and founder of the SWC at Los Angeles, CA, and to Rabbi Abraham Cooper, dean associate of the same center, in relation to the complaint made to the French Minister of Health.

We travelled to Bucharest for the first time between October 16 and 22, 2007. The host of our stay in the Romanian capital was Constantin Ionescu-Tirgoviste, whose kind help was essential for consulting archived documents and visits several institutions: the Medical School, the University Central Library **(Supplementary Figure 7)**, the Romanian Academy, the Institute of Nutrition and Metabolic Diseases “Nicolae Paulescu” **(Supplementary Figure 8)** and NC Paulescu House-Museum. With the help of translators, we had the opportunity to meet and interview several people who were able to provide us with relevant information about the biography of Paulescu [1; pp. 634-669].

The house where Palescu spent the last two decades of his life in the company of his sister Elena, was inhabited in 2007 by Dan Angelescu, son of Dr. Constantin Angelecu, Paulescu's nephew and scientific collaborator [1; pp. 644-649].

**Supplementary Material (4).**

**List of documents written by NC Paulescu revealing his religious, nationalist and political thoughts**

| **List of documents (chronological order)**  Paulescu, N. C. (1903). La génération spontanée et le darwinisme devant la méthode expérimentale. *J. Med. Interne*, 7, 203-210. [Spontaneous generation and Darwinism in the face of experimental method]  ___. (1908). *Finalité, matérialisme, âme et Dieu*. Paris: Librairie Bloud. [Finality, materialism, soul and God]  ___. (1908). *Physiologie philosophique: définition de la physiologie, méthode expérimentale, génération spontanée et darwinisme*. Paris: Librairie Bloud. [Philosophical physiology: definition of physiology, experimental method, spontaneous generation and Darwinism]  ___. (1910). *Fiziologia Filozofică: Instincte Sociale-patimi si conflict-remedii morale.* Bucharest. [Philosophic Physiology: Social Instincts - passion and conflict - moral remedies]  ___. (1913). *Fiziologia Filozofică: Talmudul, Cahalul, Francmasoneria.* Bucharest. [Philosophic Physiology: the Talmud, the Kahal, Freemasonry]  ___. (1924). *Fiziologia Filozofică: Sinagoga si biserica fată de pacificarea omenirii.* Bucharest: Apărareă Nătională. [Philosophic Physiology: Synagogue and the church in the face of the pacification of mankind]  ___. (1924). *Complot jidano-francmasonic improtiva neamului Romănesc.* Bucharest: Cartea Medicală. [Judeo-Masonic plot against the Romanian nation]  ___. (1926). *Spitalul, Coranul, Talmadul, Cahalul, Francmasoneria*. Bucharest: Editura Vicovia. [Philosophic Physiology: The Hospital, the Koran, the Talmud, the Kahal and Freemasonry]  ___. (1927). *Jidani si Alcoolismul.* Bucharest. [Jews and Alcoholism]  ___. (1927). *Talmucirea apocalipsului, soarte viitare a jidănimii.* Bucharest. [The Talmud of the Apocalypse, the future fate of Judaism]  ___. (1928). *Degenerea rasei jidanesti.* Bucharest: Fundatia Culturală Regel Milai I. [Degeneration of the Jewish race]  ___. (1928). *Desfrâul Jidanilor*. Bucharest. [The Jews' Unraveling] |
| --- |

**Supplementary Material (5).**

**Legends of Figures**

**Supplementary Figure 1.** First 2 pages of the IDF News Bulletin, vol XIV (2): Editorial notes and Report of the Special Committee to present a written summary of work leading up to the discovery of insulin. Personal Documents of NC Paulescu. (Courtesy of Dan Angelescu†) [12]

**Supplementary Figure 2.** Message from F.G. Young, President of IDF: Report of the Special Committee set up to present a written summary of work leading up to the discovery of insulin. News Bulletin of the IDF; vol XIV (2), 1971. Personal Documents of NC Paulescu. (Courtesy of Dan Angelescu†) [12].

**Supplementary Figure 3**. Excerpts from the press release of the Romanian Jewish Community (Nicolae Cajal). ISRO-Press Newsletter Excepts. Newsletter 287. Sunday, August 31, 2003. Public domain [1: p. 609]

**Supplementary Figure 4.** Letter from the presidency of the Romanian Academy to the IDF presidency

requesting recognition of Paulescu's scientific merits, outside the sociopolitical debate for his anti-Semitism (September 20, 2003). Personal Archive of NC Paulescu. Courtesy of Dan Angelescu† [1: pp. 610-612]

**Supplementary Figure 5. Left**: Letter from Prof. G. Slama, Director of the Diabetes Department of the Hôtel Dieu, published in *The Lancet*, justifying the cancellation of the events held in honor of NC Paulescu (October 25, 2003). **Right:** Letter to *The Lancet* signed by Georg Alberti and Pierre Lefèbvre (December, 2003) regarding “the Paulescu case”

**Supplementary Figure 6.** Program of the International Symposium of Experts *Who discovered insulin?* Heldin Delphi (Greece), September 8th, 2005, on the occasion of the 41st Annual EASD Meeting.

**Supplementary Figure 7. Left:** Carol Davila University of Medicine and Pharmacy (photo taken by the authors in 2007). Partial view of the Central Library at the Foundation of the Bucharest University with King Carol I statue at the front. Author of the photography, Marian Nedelcu; date, January 8, 2014

**Supplementary Figure 8**. The National Institute of Diabetes, Nutrition and Metabolic Diseases “Nicolae Paulescu”. In the entrance patio to the institute, the commemorative plaque to honor Lanceraeaux and Paulescu and both statues have been displayed after been rejected their instalation at the Hôtel Dieu in Paris (photos taken by the authors in October 2007)

**Supplementary Material (6).**

**Acknowledgments.**

Many people have contributed in different tasks to the final result of this research. Profs. J.L. Barona-Vilar and Àlvar Martínez Vidal (Department of History of Science, Medicine and Scientific Communication, Univ. of Valencia-López Piñero, Research Institute) and Dr. C. Pérez-Aguado (Fundació DIABEM) provided insightful comments to improve the manuscript draft. Prof. Constantin Ionescu-Tirgoviste, Carol Davila University of Medicine and Pharmacy (Bucharest), member of the Romanian Academy and former director of the Institutul National de Diabet, Nutritie si boli Metabolice “Nicolae Paulescu” made it possible to consult archives on NC Paulescu at the Romanian Academy and the Library of the Faculty of Medicine. Moreover, he accompanied us on the visit to the house-museum of the Romanian physiologist, facilitated free access to the existing personal documents of NC Paulescu, kept in the house-museum by Dan Angelescu, organized the interviews with G. Brautescu and Serban Micoveanu, and in 2010 gave us copies of the three volumes of the 30th edition, revised by himself, of Paulescu's Traité de Physiologie Médicale. Dan Angelescu (died 2013) surprised us in October 2007 with the wonderful gift, one copy of the first edition of volume II of the Traité de Physiologie Médicale, published in 1916, and copies of the first edition of all four volumes of the Traité de Médecine by Lancereaux and Paulescu books that we have registered in the archive of Fundació DIABEM Library of history of medicine. Professor Nicolae Hancu, former Director of the Department of Endocrinology, Metabolism and Nutrition at the University of Cluj-Napoca, former President of the Romanian Diabetes Society and member of the Romanian Academy, repeatedly invited us to participate in the annual congress of the Romanian Diabetes Society and enlightened us on the organization of clinical and research assistance on diabetes in Romania. Professor Maria Moța, Head of the Department of Diabetes at Craiova University of Medicine and Pharmacy during her tenure as President of the Romanian Diabetes Society, gave us similar help and invited us to present our views on the controversy and priority of discovery of the antidiabetic hormone.

Jose Luiz Medina^Ϯ^, Emeritus Professor at the University of Porto and former President of the Portuguese Society of Diabetology, and Doctors Luis Gardete-Correia and Jose Manuel Boavida, co-directors of the Association for the Protection of Diabetic Patients of Portugal (APDP), warmly welcomed and invited us to present different stages of the progress of our research on the discovery of the antidiabetic hormone.

Ilie Simon, retired professor from the University of Bucharest, and his daughter Cristina, both living in Barcelona, ​​altruistically volunteered to translate documents related mainly to Paulescu's sphere of social and political actions. Doctor Sorin Mucanu, currently Head of the Surgery Department of the General Hospital of Catalonia, and his wife Raluca, an ophthalmologist, selflessly collaborated in the initial stage of our activities with the translation of Paulescu's scientific and sociopolitical documents written in Romanian. Ruxandra Lungu, a translator and terminologist, also contributed with the translation of texts of political nature from Paulescu and other influential academics in Romanian society.

Aldoiu Mircea (Official Library of Stat pentru Invenții şi Mãrci) provided us with a copy of the documentation on the pancreina patent. In the nearby scenario, Ms. Teresa Mas, from the Historical Archive of the Hospital de Sant Pau and the Library of the Fundació Universitat Autònoma de Barcelona, as well as Ms. Eulàlia Brugués, specialist in information systems and graduate in Pharmacy, from the staff of Fundació DIABEM, a private non-profit institution, have contributed to the acquisition of documents from various countries and to digitizing and preparing reports in accordance with new technologies.
