# Supplementary material for: On the occasion of the centennial of the Nobel Prize in Physiology or Medicine, 1923: Nicolae C. Paulescu—between scientific creativity and political fanatism
Source: Acta Diabetol. 2023 Jul 5;60(11):1513–30. doi: 10.1007/s00592-023-02136-6 (PMC10520201; doi:10.1007/s00592-023-02136-6)

## Slide 1
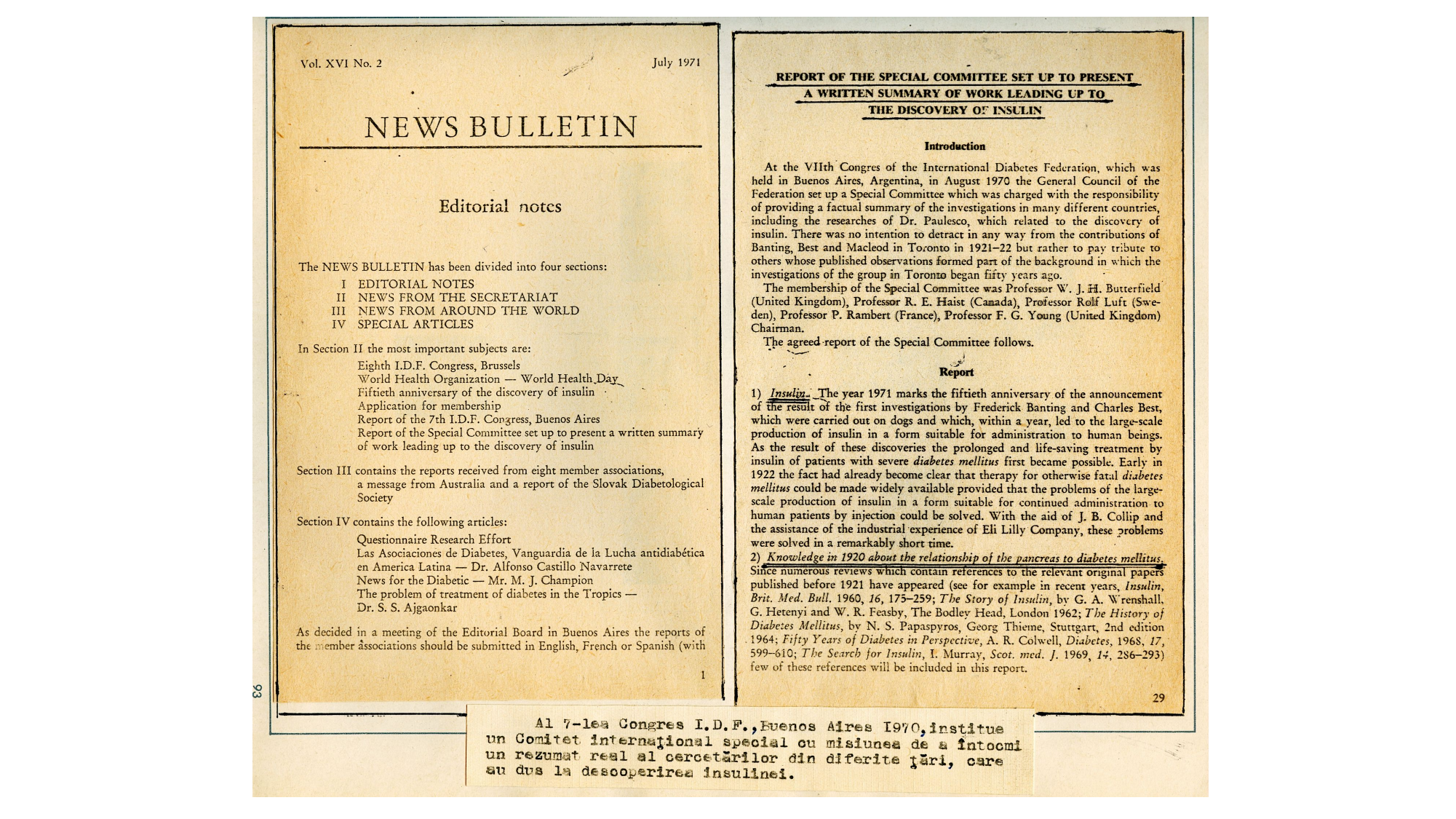

## Slide 2
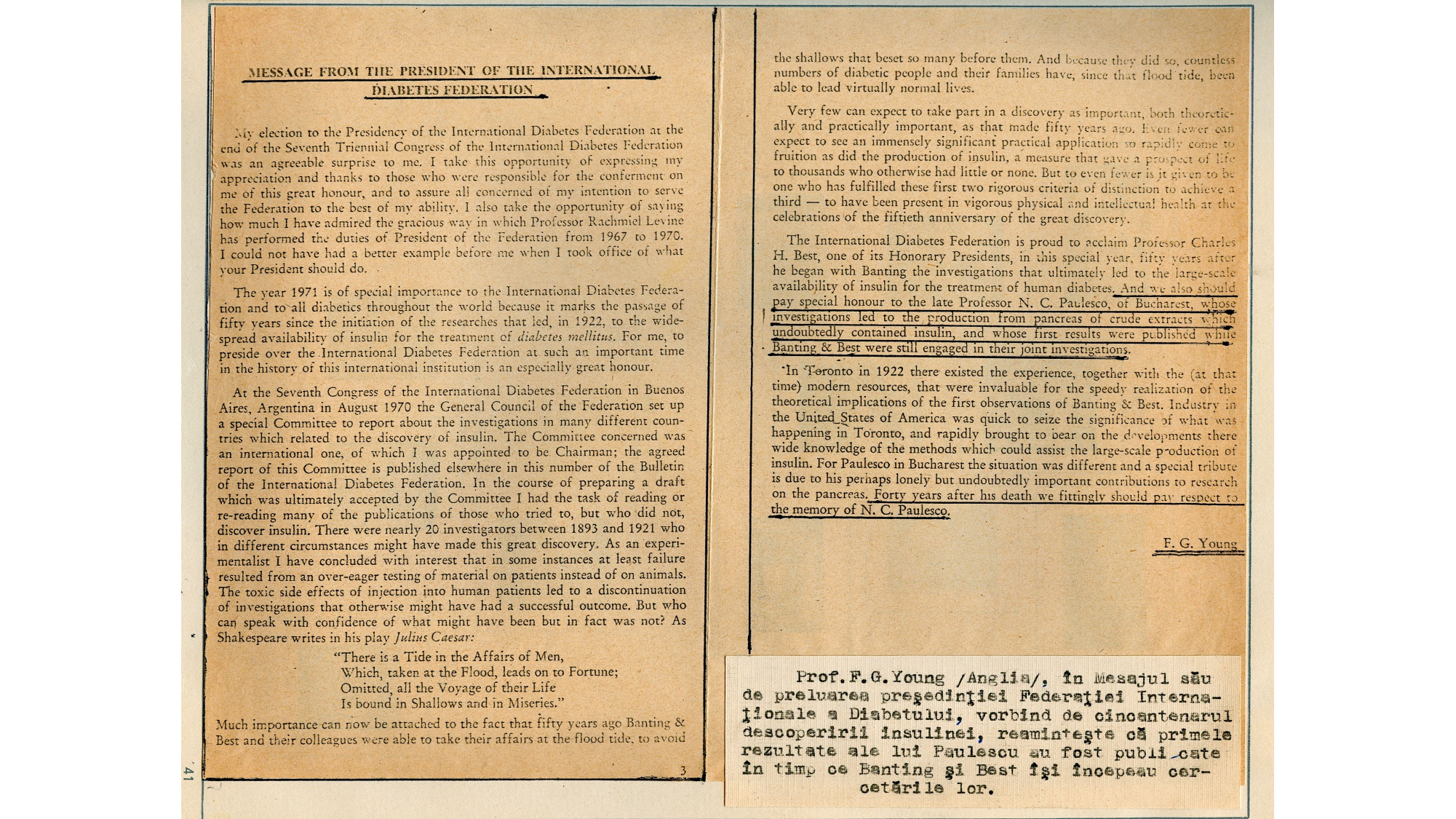

## Slide 3
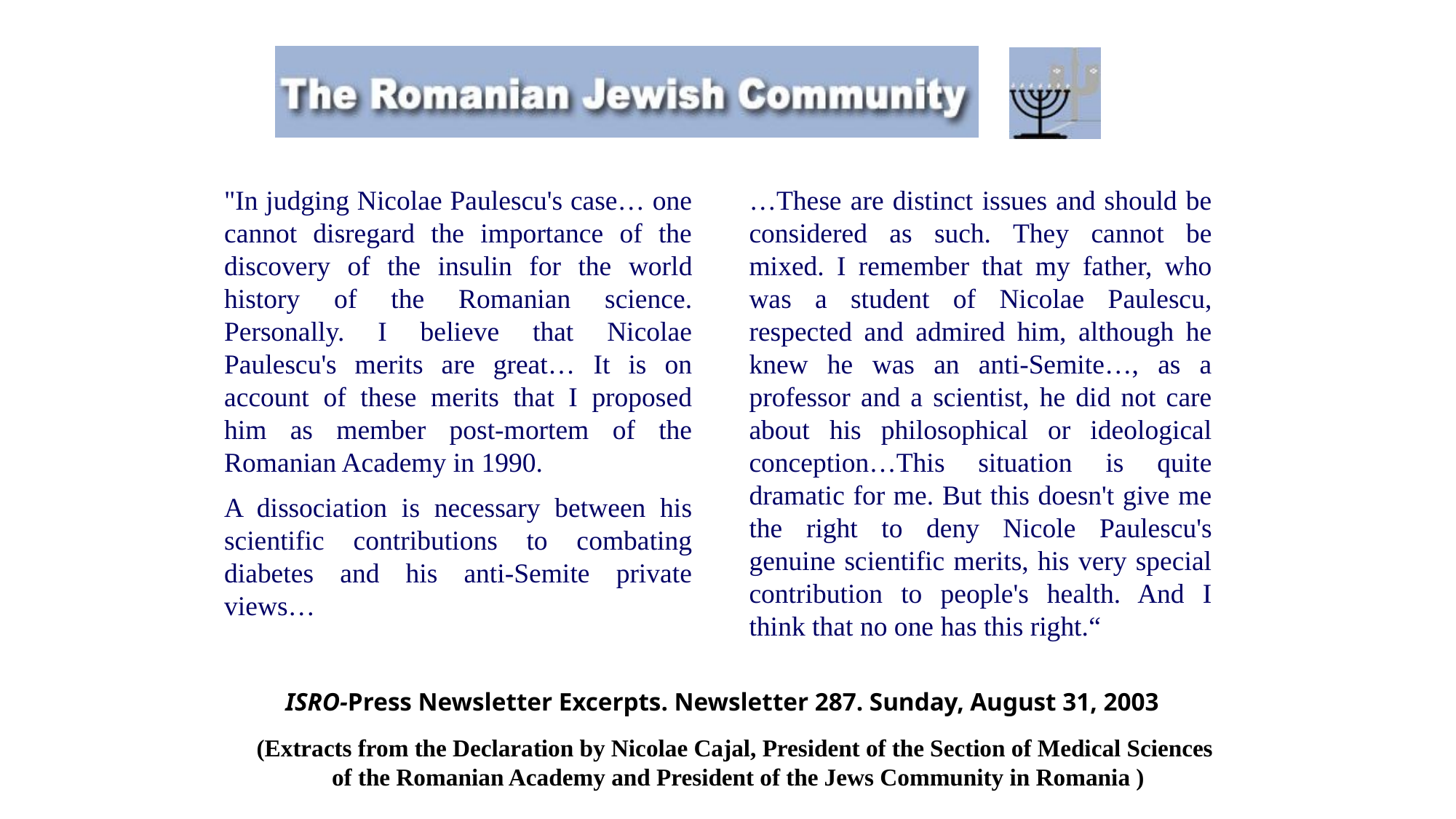

"In judging Nicolae Paulescu's case… one cannot disregard the importance of the discovery of the insulin for the world history of the Romanian science. Personally. I believe that Nicolae Paulescu's merits are great… It is on account of these merits that I proposed him as member post-mortem of the Romanian Academy in 1990.
A dissociation is necessary between his scientific contributions to combating diabetes and his anti-Semite private views…
…These are distinct issues and should be considered as such. They cannot be mixed. I remember that my father, who was a student of Nicolae Paulescu, respected and admired him, although he knew he was an anti-Semite…, as a professor and a scientist, he did not care about his philosophical or ideological conception…This situation is quite dramatic for me. But this doesn't give me the right to deny Nicole Paulescu's genuine scientific merits, his very special contribution to people's health. And I think that no one has this right.“
ISRO-Press Newsletter Excerpts. Newsletter 287. Sunday, August 31, 2003
(Extracts from the Declaration by Nicolae Cajal, President of the Section of Medical Sciences
of the Romanian Academy and President of the Jews Community in Romania )

## Slide 4
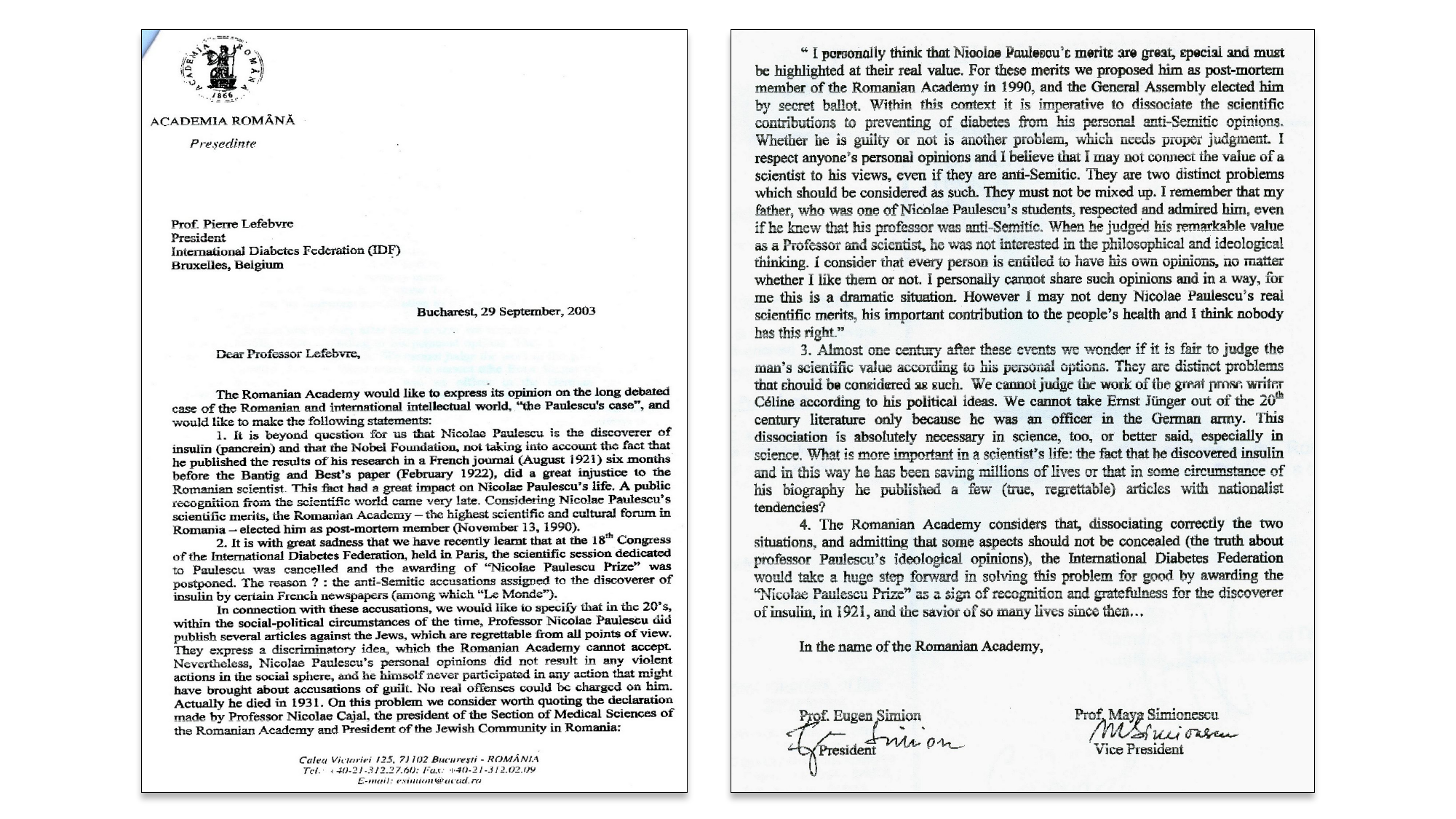

## Slide 5
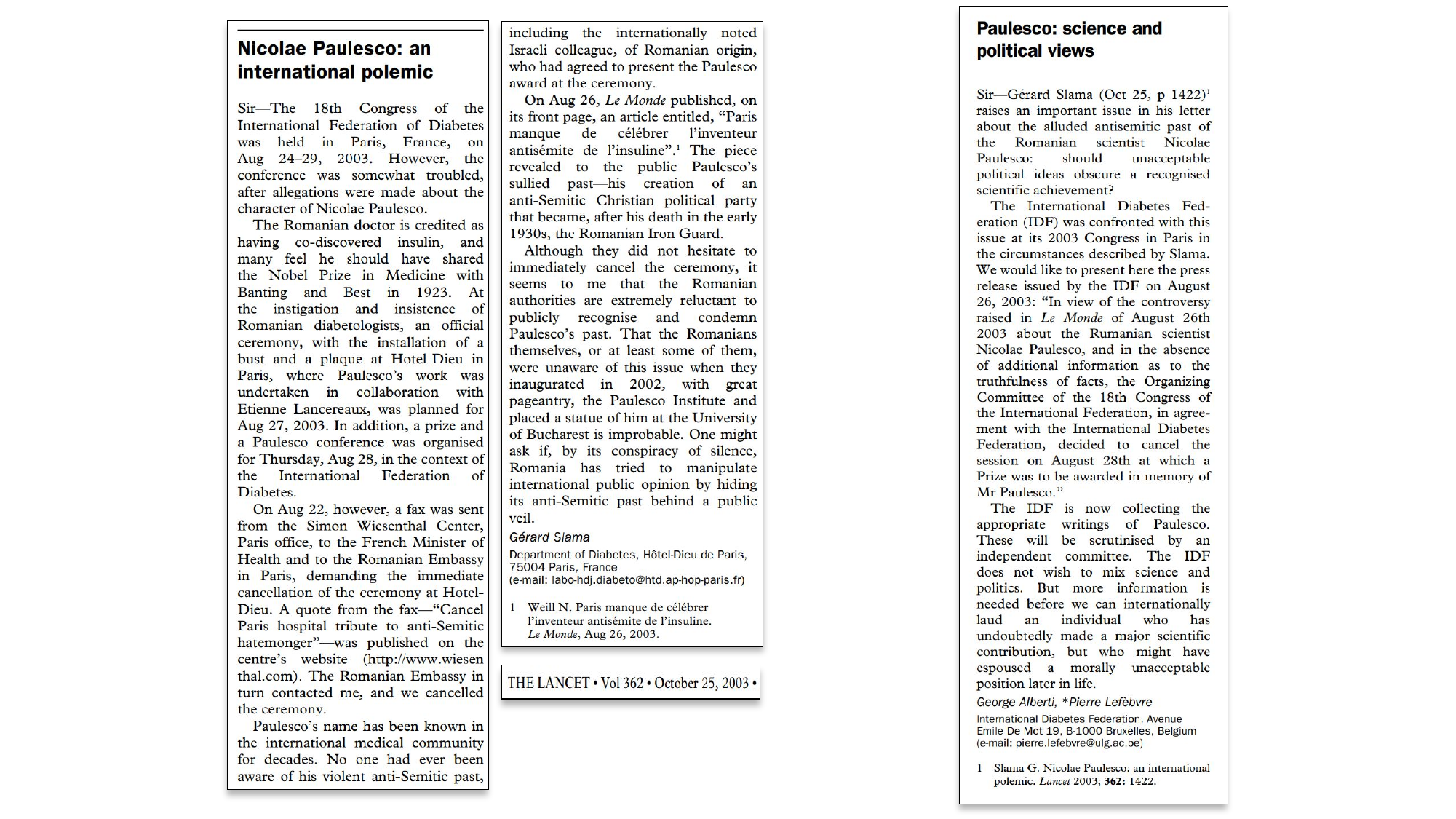

## Slide 6
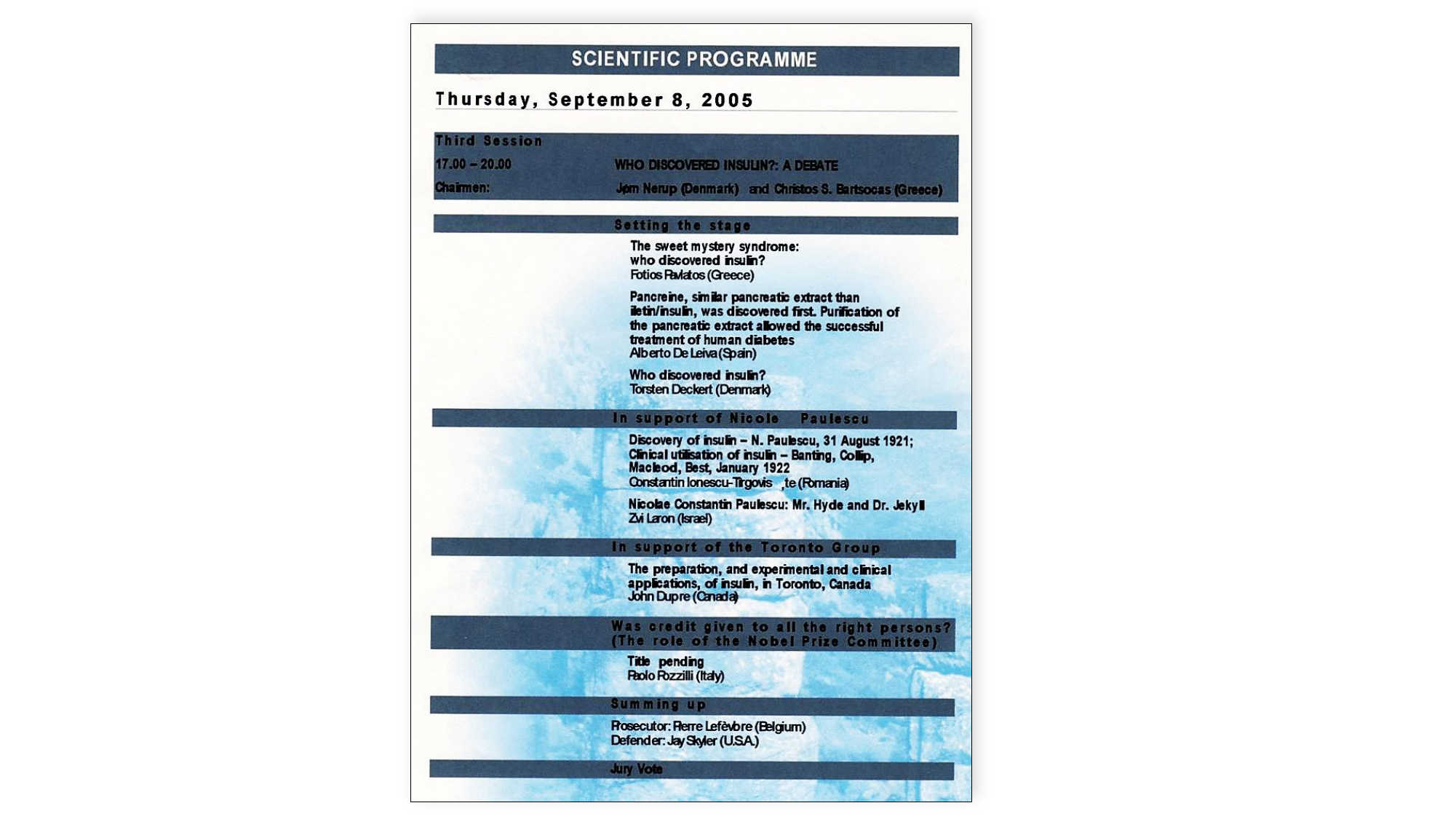

## Slide 7
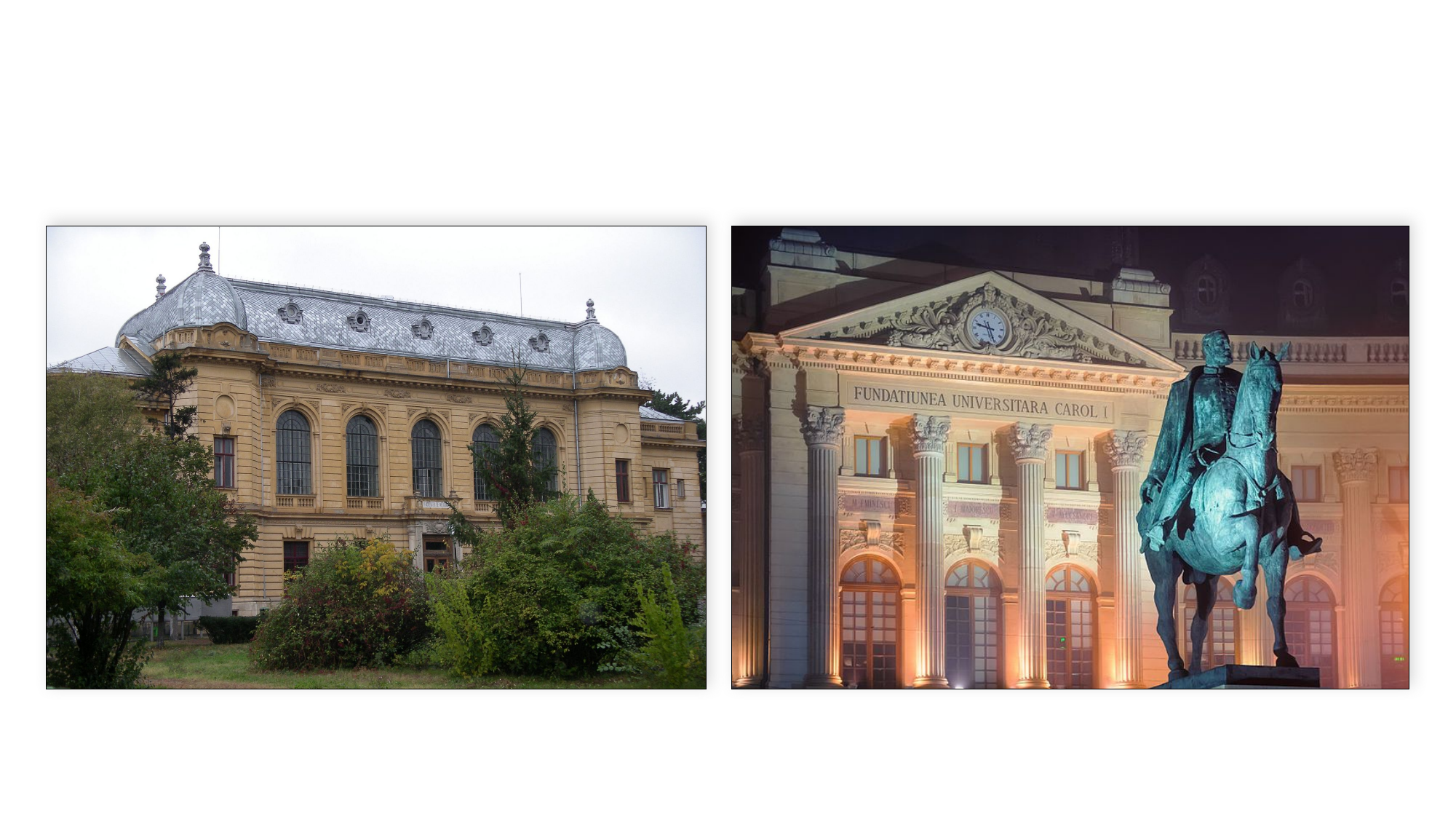

## Slide 8
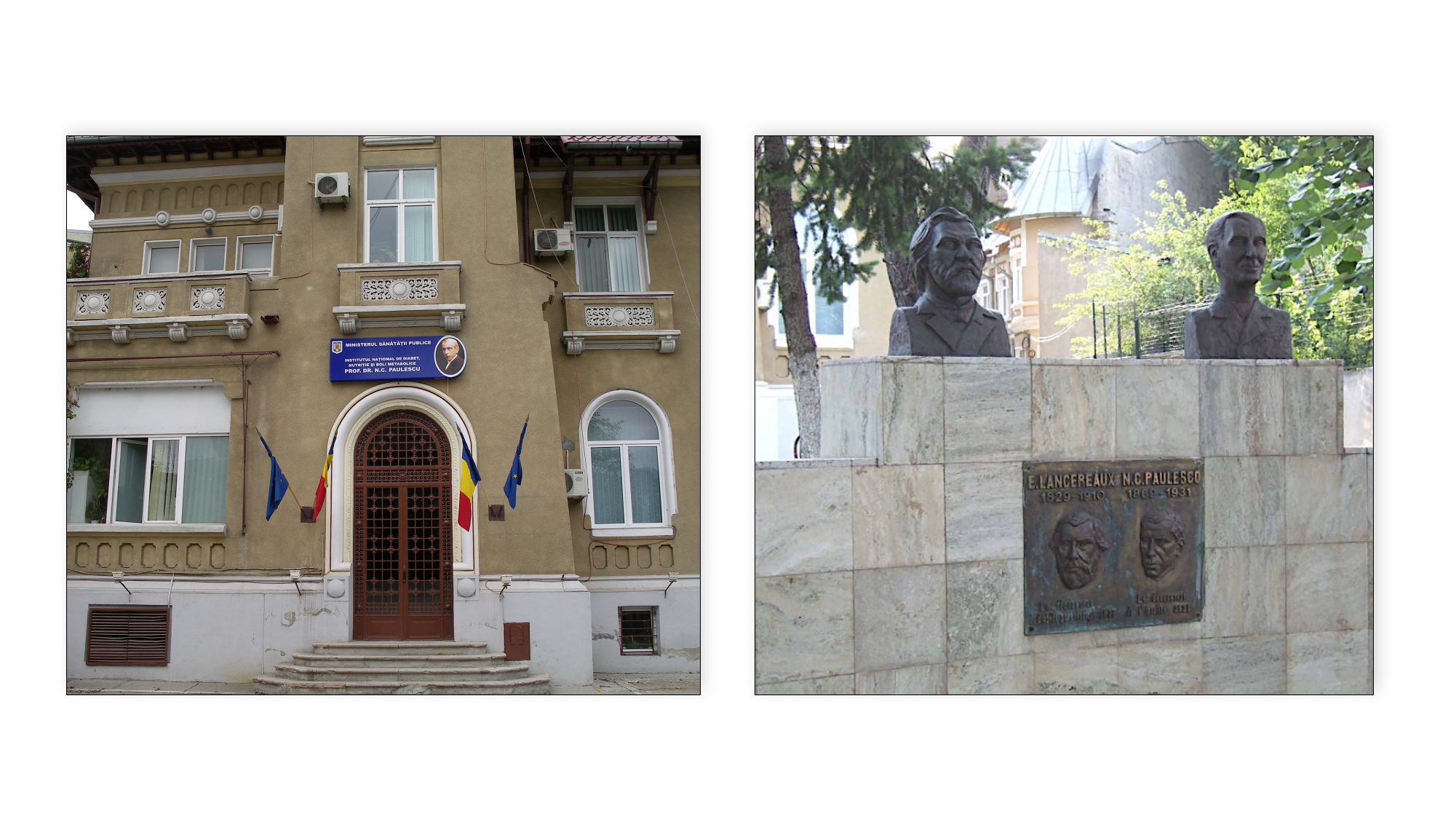

Supplement: Supplementary file 2 — Supplementary file2 (PPTX 53111 KB) [file 592_2023_2136_MOESM2_ESM.pptx]
